# Molecular genetic analysis using targeted NGS analysis of 677 individuals with retinal dystrophy

**DOI:** 10.1038/s41598-018-38007-2

**Published:** 2019-02-04

**Authors:** Cathrine Jespersgaard, Mingyan Fang, Mette Bertelsen, Xiao Dang, Hanne Jensen, Yulan Chen, Niels Bech, Lanlan Dai, Thomas Rosenberg, Jianguo Zhang, Lisbeth Birk Møller, Zeynep Tümer, Karen Brøndum-Nielsen, Karen Grønskov

**Affiliations:** 1 Kennedy Center, Department of Clinical Genetics, Rigshospitalet, University of Copenhagen, Glostrup, DK2600 Denmark; 2 BGI-Shenzhen, Shenzhen, 518083 China; 3 China National GeneBank, BGI-Shenzhen, Shenzhen, 518120 China; 4 Department of Laboratory Medicine, Division of Clinical Immunology and Transfusion Medicine, Karolinska University Hospital Huddinge, Stockholm, S141 86 Sweden; 5 Department of Ophthalmology, Rigshospitalet-Glostrup, University of Copenhagen, Glostrup, DK2600 Denmark; 6 Department of Clinical Medicine, Faculty of Health and Medical Sciences, University of Copenhagen, Copenhagen, Denmark

## Abstract

Inherited retinal diseases (IRDs) are a common cause of visual impairment. IRD covers a set of genetically highly heterogeneous disorders with more than 150 genes associated with one or more clinical forms of IRD. Molecular genetic diagnosis has become increasingly important especially due to expanding number of gene therapy strategies under development. Next generation sequencing (NGS) of gene panels has proven a valuable diagnostic tool in IRD. We present the molecular findings of 677 individuals, residing in Denmark, with IRD and report 806 variants of which 187 are novel. We found that deletions and duplications spanning one or more exons can explain 3% of the cases, and thus copy number variation (CNV) analysis is important in molecular genetic diagnostics of IRD. Seven percent of the individuals have variants classified as pathogenic or likely-pathogenic in more than one gene. Possible Danish founder variants in *EYS* and *RP1* are reported. A significant number of variants were classified as variants with unknown significance; reporting of these will hopefully contribute to the elucidation of the actual clinical consequence making the classification less troublesome in the future. In conclusion, this study underlines the relevance of performing targeted sequencing of IRD including CNV analysis as well as the importance of interaction with clinical diagnoses.

## Introduction

The introduction of next generation sequencing (NGS) has improved molecular genetic diagnosis of genetically heterogeneous conditions substantially. This is also true for inherited retinal diseases (IRDs) which are associated with sequence variations in more than 150 genes (https://sph.uth.edu/retnet/). IRDs encompass a heterogeneous group of retinal disorders with degeneration of photoreceptors, and are considered one of the leading causes of visual impairment in children and young individuals^1^. Retinitis pigmentosa (RP) is the most frequent clinical diagnosis of the IRD specific diagnostic subgroups with a worldwide prevalence of 1:4000^2^. Diagnoses include both syndromic and non-syndromic conditions. IRD can be inherited as autosomal recessive (AR), autosomal dominant (AD), X-linked (XL) and mitochondrial traits; furthermore, digenic inheritance has been reported in rare cases^3^.

A genetic diagnosis is not only important to improve the genetic counselling of the families and for prognostic reasons but also for identifying patients that might benefit from new candidate treatments such as gene therapy, where promising results are being reported in numerous clinical trials worldwide^4^.

In this study, we present genetic investigation of a cohort of 677 individuals residing in Denmark and clinically diagnosed or suspected with IRD. Coding regions together with 20 bp flanking intronic sequence and untranslated regions (UTRs) of 125 genes previously shown to be associated with IRD, were analyzed using panel-based NGS.

We report all variants that could be potentially disease causing; it is noteworthy that the number of rare variants per individual requires meticulous scrutiny combining several tools; and a close collaboration between clinical and laboratory medicine is mandatory.

## Results

### Patient characteristics

**Figure 1:**
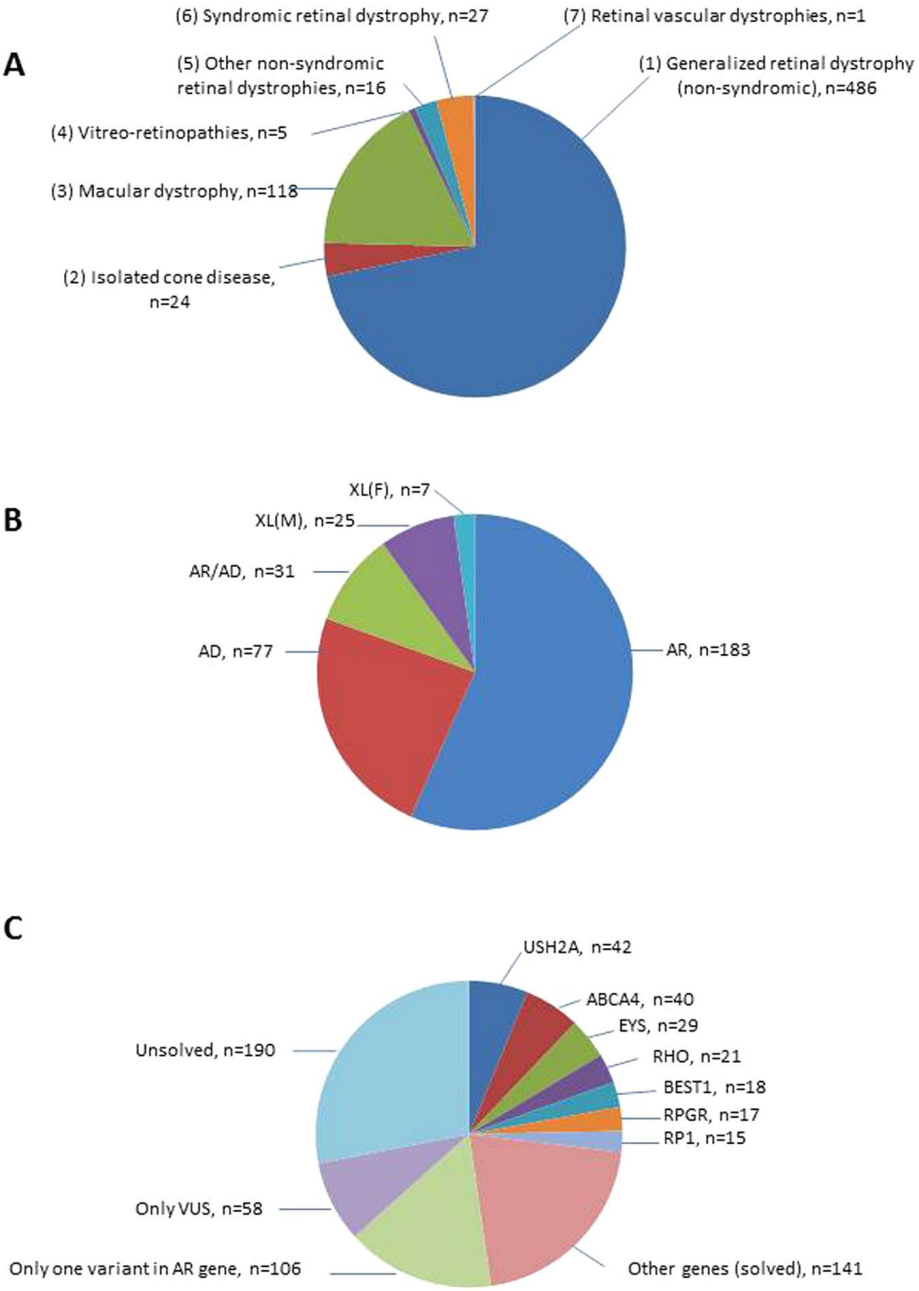
(**A**) Distribution of 677 individuals in seven groups based on clinical diagnosis. The number in brackets refer to the clinical group, and n = number of individuals. (**B**) Inheritance based on genetic findings in 323 individuals with a molecular genetic diagnosis; n = number of individuals. (**C**) Mutational spectrum of variants identified in 677 individuals with IRDs; n = number of individuals.

Patients clinically suspected of having a retinal dystrophy were selected for the targeted analysis based on evaluation of available clinical records (clinical diagnosis, clinical history, fundus changes, OCT imaging, ERG, visual acuity, visual fields, family history). A total of 677 individuals were investigated by a targeted NGS panel consisting of 125 genes. Samples have been collected to a clinical biobank over many years. The clinical diagnoses were divided into seven groups (Table S1, Fig. 1A). The group “Generalized retinal dystrophies” which includes RP and cone-rod dystrophies were by far the most common clinical diagnosis (72%), followed by “Macular dystrophy” which includes Stargardt disease (17%)^5^. The distribution of clinical diagnoses is biased due to several previous research projects of specific diagnostic subgroups and diagnostic efforts during the years. Individuals with one of following clinical diagnoses: Stargardt disease^6^, achromatopsia^7^, Usher syndrome^8^, Bardet-Biedl syndrome^9^, Best vitelliform macular dystrophy^10^, choroideremia^11^, Leber congenital amaurosis^12^, XL and AD retinitis pigmentosa^13^, congenital stationary night blindness^14^, cone dystrophy and Åland eye disease^15^ were included in previous research projects, and are therefore represented by lower numbers than might be expected.

### NGS panel

The 125 genes in the panel are known to be associated with: retinitis pigmentosa (41%), Bardet-Biedl syndrome (14%), cone-rod dystrophy (12%), Leber congenital amaurosis (10%), Usher syndrome (7%) and congenital stationary night blindness (3%) (some genes cause more than one eye disorder); Other genetic retinal diseases account for a small proportion in the panel (Table S2). 70% of the genes are associated with an AR inheritance pattern, 20% with an AD inheritance pattern and 5% with a XL inheritance pattern; furthermore, two genes (*PRPH2* and *ROM1*) have been reported to be involved in digenic inheritance (Table S2).

We obtained on average 1156.22 Mb per individual that mapped to the target region. The mean depth of the target regions of all samples was 533X (range from 181X to 1587X) and the average coverage of at least 4X and 20X was 98.27% and 97.29%, respectively (Table S3).

Variants were filtered based on quality, frequency and function. A total of 7440 rare variants (4030 SNVs and 3410 indels) remained with a mean number of 9.24 rare variants per individual (range from 2 to 29). The reference sequence is GRCh37/hg19 and the exons are numbered according to reference sequences as listed in Table S2.

### Sequence variations

**Figure 2:**
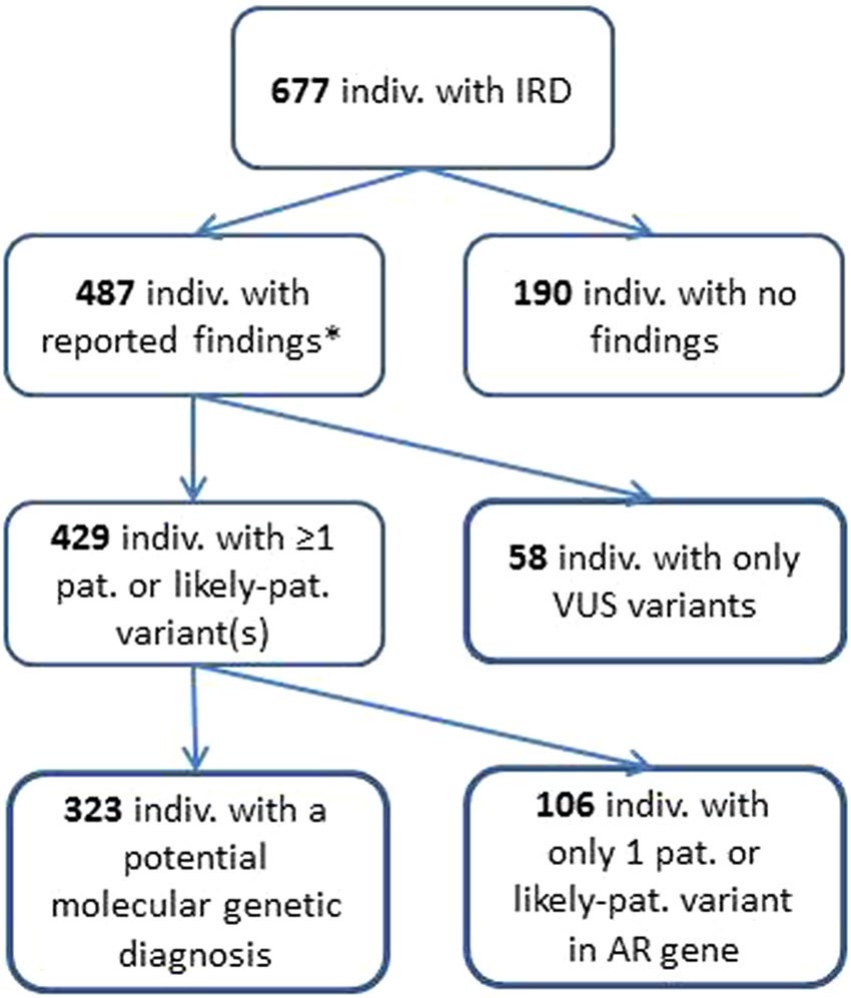
Flow of individuals. A schematic representation showing the outcome of the 677 individuals with IRD participating in the study. *Supplementary Table 4. Indiv: individuals.

We report all variants considered to be potentially disease causing, including variants classified as VUS (variant of unknown clinical significance) when it was estimated that they could affect the phenotype (Table S4). Variants were classified according to the American College of Medical Genetics and Genomics (ACMG) classification. A total of 806 variants, 532 unique variants, are reported; of these 187 were novel at the time of manuscript submission. Potential disease causing variants were found in 487 individuals while in 190 individuals we found no variants to report (flow of individuals are shown in Fig. 2). Pathogenic or likely-pathogenic variants was identified in 429 out of 677 individuals (63%), while 58 individuals out of 677 (9%) had either one variant classified as VUS in a gene with AD or XL inheritance, or two variants classified as VUS in a gene with AR inheritance. In 323 individuals (48%) a plausible molecular genetic diagnosis could be established while in 106 individuals (16%) only one pathogenic or likely-pathogenic variant in an AR gene was identified. The study revealed unprecedented complexity in the interpretation of the sequence variants as 7% (47/677) of individuals harbored pathogenic or likely-pathogenic variants in more than one gene.

The sequence variations are distributed across 81 genes; 59 genes with AR inheritance (n = 183), 14 genes with AD inheritance (n = 77), four with XL inheritance (n = 32) and four genes known with both AR and AD inheritance (n = 31) (Fig. 1B). Numbers in brackets indicate the number of individuals. Of the 323 individuals with a plausible molecular genetic diagnosis, the most frequently mutated genes were *USH2A* (n = 42, 6%), *ABCA4* (n = 40, 6%), *EYS* (n = 29, 4%), *RHO* (n = 21, 3%), *BEST1* (n = 18, 3%), *RPGR* (n = 17, 2%) and *RP1* (n = 15, 2%). Variants in these seven genes could explain more than half of the solved cases (182 out of 323, 56%) (Fig. 1C). Variants in 22 genes solved only one or two cases each, demonstrating the necessity of a large gene panel for IRD analysis. Furthermore, in 47 individuals, sequence variations classified as either pathogenic or likely-pathogenic were detected in more than one gene (Table S4).

CNV analysis was performed using NGS data, and indications of a deletion or duplication were confirmed with another method (MLPA (Multiplex Ligation-dependent Probe Amplification), qPCR (quantitative PCR analysis) or chromosome microarray). Furthermore, all samples heterozygous for a pathogenic or likely-pathogenic sequence variant in *ABCA4*, *USH2A* or *EYS* were analyzed by MLPA to search for deletions and duplications which revealed duplications in *EYS* (case 126) and *USH2A* (case 297). In total, this strategy detected 17 deletions and seven duplications resulting in a molecular genetic diagnosis in 17 cases (Table S5).

### Danish disease associated variants and other disease associated founder variants

A novel disease associated variant, c.232del in *EYS*, was detected in 11 unrelated individuals. Using the surrounding SNPs in *EYS* we detected a common haplotype spanning 1.9 Mb (chr6:64,431,148-66,415,690, GRCh37/hg19). Likewise, a novel disease associated variant, c.2690_2695del in *RP1*, was detected in five unrelated individuals. Analysis of the surrounding SNPs demonstrated a common haplotype spanning 14 kb (chr8:55,528,953-55,543,161, GRCh37/hg19) (Table S6).

## Discussion

Targeted NGS analysis is a valuable method for molecular genetic diagnostics of IRDs as also supported by several previous studies^16–18^. Our detection rate of 48% is relatively low compared to other studies; however, this may be biased as our study population was derived from clinical biobank samples that had been extensively investigated over the years in several research projects and diagnostics efforts, resulting in many solved cases not included in the present study.

A substantial number of individuals were found with only one pathogenic or likely-pathogenic sequence variant in a gene with AR inheritance and clinical symptoms that support the genetic diagnosis. This type of incomplete genetic diagnosis (missing heritability) is a well-known phenomenon. The missing genetic alterations might be found in regulatory regions or deep intron sequences, but also frequent hypomorph variants which are filtered out during data analysis or combinations of such in a haplotype could be an explanation of the missing heritability. Furthermore, given the high frequency of AR inherited retinal dystrophy, it is of course possible that a yet unknown cause is responsible for the eye disorder. Digenic inheritance has also been observed for retinal dystrophies, however, we did not observe any obvious signs of this. Whole genome sequencing combined with segregation analysis and functional studies could solve some of these cases in future studies.

In 9% of individuals, variants classified as VUS (either one in a gene with AD or XL inheritance, or two in a gene with AR inheritance) were identified where the clinical symptoms were supportive. These variants are also reported in this study since the classification of variants may change over time, and ideally, variants should be classified as either pathogenic (disease associated) or benign; but currently many variants are classified as VUS and the only way forward is to report the variants and combine studies.

The most genetically heterogeneous group was the RP group in which causative variations were found in 32 genes, all of which are known to be associated with RP. The diagnosis Stargardt disease was almost exclusively associated with variants in *ABCA4*. However, we also found one individual with p.(Arg373Cys) missense variant in *PROM1* which has previously been reported to be associated with Stargardt disease. Notably, we found two individuals with truncating variants in *CRX*. Recently, a nonsense variant in *CRX* was found in an individual with adult onset macular dystrophy^19^.

CNV analysis revealed a total of 24 deletions and duplications. In some cases their pathogenecity was questionable, while in others they were plausible explanations. It is obvious that CNV analysis is important in molecular genetic diagnosis of IRD, both shown in the present study and by others in previous studies^20^.

Several individuals had variants in more than one gene. Six individuals (cases 53, 135, 138, 180, 194 and 202) had sequence variations in two genes that potentially both could be the cause of IRD (Table S4). In case 53 (a male), a pathogenic change in *BEST1* explains the Best vitelliform macular dystrophy phenotype, however, a truncating variant in *RPGR* would also be expected to be causative. Case 135 was found to be compound heterozygote for variants in both *EYS* and *CDH23*; he was diagnosed clinically with RP and had a mild hearing impairment. It could be speculated that the variants in *EYS* caused the RP while variants in *CDH23* caused the hearing impairment. It is noteworthy though, that analysis restricted to genes known to be associated with Usher syndrome based on the clinical symptoms would have revealed only the *CDH23* variants while the *EYS* variants would have remained undetected. Case 138 with a clinical diagnosis of congenital stationary night blindness was homozygous for likely-pathogenic variants in both *GRK1* and *TRPM1*. Both genes are associated with congenital stationary night blindness with AR inheritance. Likewise, case 180 had variants in both *PRPF31* and *ELOVL4*; both genes are associated with RP with AD inheritance. Case 194 had deletions in two genes (*PRPF31* and *IMPDH1*); both genes show AD inheritance, and both genes are associated with RP. Case 202 had a clinical RP diagnosis and we detected a *PRPF31* splice variant and two *ABCA4* variants. Segregation analysis was unfortunately not possible in these cases.

In conclusion, this study shows that targeted NGS is an effective method for establishing a molecular genetic diagnosis of IRDs. CNV analysis should be part of the strategy. Using a large gene panel is a prerequisite since a similar phenotype can be caused by many genes, and variants can be present in more than one gene, which is important knowledge if the result is to be used in treatment purposes and for genetic counseling. We report sequence variants both from individuals who received what is considered to be a molecular genetic diagnosis with results consequently reported to the clinician, as well as from those receiving a partly molecular genetic diagnosis. This information is valuable in the further classification of variants, and in phenotype-genotype correlation studies.

## Methods

### Patients and clinical evaluation

The investigated cohort comprises 677 unrelated individuals residing in Denmark with a clinical diagnosis of IRD. Ages of the individuals ranged from 4 to 100 years with a mean age of 53.3 years and a median age of 55 years. 493 individuals without a previous molecular genetic diagnosis and with a DNA sample in the clinical biobank at Kennedy Center, Rigshospitalet, were identified from the Retinitis Pigmentosa Registry and from assessment of clinical records based on diagnosis codes related to any form of retinal dystrophy at the Department of Ophthalmology, Rigshospitalet. The Retinitis Pigmentosa Registry is a nation-wide register in which all individuals residing in Denmark with a diagnosis of a generalized retinal dystrophy born after 1850 has been registered^5,21^. Furthermore, 184 individuals were enrolled during the study. Most participants were of Danish origin. Unfortunately, due to the fact that samples have been collected over many years, segregation analysis was in most cases not possible.

The project was approved by The National Committee on Health Research Ethics (Denmark). The project was performed according to the Declaration of Helsinki and approved by the Regional Ethics Committee. Written informed consent was obtained from all the participants and if under the age of 18 from a parent or legal guardian, before the molecular genetic testing.

### Gene selection and target enrichment

We selected 125 genes that were reported to be associated with inherited retinal disorders according to RetNet database (https://sph.uth.edu/retnet/), OMIM (the Online Mendelian Inheritance in Man, http://www.omim.org/) and literature searching (Table S2). The custom NimbleGen SeqCap Target Enrichment System (NimbleGen, Madison, WI, USA) was designed to capture and enrich all coding exons, 5-/3-UTR regions and 20 bp flanking intronic regions. The size of the target region was 2.07 Mb and the coverage rate was 95%.

Individuals who were heterozygous for an *ABCA4* or *USH2A* pathogenic or likely-pathogenic variant were analyzed for deep intron variants reported to be associated with disease. For investigation of *ABCA4* intronic regions, chr1:94,483,922-94,484,082 and chr1:94,493,000-94,492,973 were amplified using primer set ABCA4-V1-3-FH acccactgcttactggcttatcGGGATCATTATGACATCAACCCC and ABCA4-V1-3-RH gaggggcaaacaacagatggcCTCCATAGGCTCAGAGATCCC and primer set ABCA4-V4-5-FH acccactgcttactggcttatcACACCATGTAGGTAGGCTTGG and ABCA4-V4-5-RH gaggggcaaacaacagatggcAGGGATCCCAAAAGAAGGAC^22^ respectively, followed by Sanger sequencing. For *USH2A*, primer set USH2A-intron40-FH acccactgcttactggcttatcAGCTTCCTCTCCAGAATCACA and USH2A-intron40-RH gaggggcaaacaacagatggcGGTTTTCATCTGGGTCTTGCA were used for PCR amplification, followed by Sanger sequencing. Sequences in lower case letters were used as tags for Sanger sequencing. In addition all individuals with a clinical diagnosis of Leber congenital amaurosis were investigated for the *CEP290* c.2991 + 1655A > G variant using primer set CEP290-intron26-FH acccactgcttactggcttatcGGTTCAGGCCGTTCTCCT and CEP290-intron26-RH gaggggcaaacaacagatggcCACATGGGAGTCACAGGGTA for PCR amplification, followed by Sanger sequencing.

### Next-generation sequencing and bioinformatics

Genomic DNA was extracted from peripheral blood using standard protocols. The enriched DNA libraries were sequenced using the Illumina HiSeq 2000 (494 patients) or HiSeq 4000 (184 patients) platforms (Illumina, San Diego, CA, USA). To optimize cost-efficient sequencing of the custom target panels, a pilot study was performed, pooling libraries from 1, 2, 5 and 8 samples. After comparing the sequencing data quality, coverage rate, genotyping concordance, sequencing depth, mismatch rate, capture specificity and total detected variants, pooling of five libraries were the optimal strategy.

Raw sequencing image files and base-calling were processed with the Illumine Pipeline and raw paired-end low quality reads and adapter sequences were removed using the SOAPnuke software (http://soap.genomics.org.cn/). The remaining high-quality reads were aligned to the human reference genome (GRCh37/hg19) using Burrows–Wheeler Algorithm (BWA) version 0.7.10^23^, with the MEM algorithm. The SAMtools (version 0.1.19)^24^ was used to sort and index SAM/BAM files and the Picard (version 1.117, http://broadinstitute.github.io/picard/) was used to mark PCR-duplicates. Local realignment and base recalibration were performed using GATK (version 3.3-0)^25^ and single nucleotide variants (SNVs) and insertions/deletions (InDels) were called with GATK HaplotypeCaller.

All identified variants were annotated using Ensembl’s VEP (Variant Effect Predictor)^26^ and variants were prioritized based on the following criteria: (1) variants not present in BGI (Beijing Genome Institute) in-house databases and with a minor allele frequency less than 1% using three public variant databases, including 1000 Genomes Project (KG, http://www.1000genomes.org/), Exome Variant Server (ESP, http://evs.gs.washington.edu/EVS/) and Exome Aggregation Consortium (ExAC; http://exac.broadinstitute.org/); there are known disease associated variants causing IRD which have a frequency above 1%, the data were screened for these separately; (2) kept all non-synonymous, small indels, frameshift, nonsense, or affect canonical splice-site donor/acceptor sites variants. Variants were verified by Sanger sequencing prior to return of results to the clinician.

### Interpretation and classification of variants

Sequence variations were classified into five categories: pathogenic (class 5), likely-pathogenic (class 4), VUS (class 3), likely benign (class 2) and benign (class 1), according to the ACMG guidelines^27^. Alamut Visual (Interactive Biosoftware, Rouen, France) was used to evaluate missense and splice variants. Alamut Visual includes the in silico tools Align GVGD^28,29^, SIFT^30^, MutationTaster^31^ and PolyPhen2^32^ for missense variants and SpliceSiteFinder-like^33,34^, MaxEntScan^35^, GeneSplicer^36^, NNSPLICE^37^ and Human Splicing Finder^38^ for splice variants.

### Deletion and duplication analysis

For targeted NGS samples, copy number analysis was performed either using the R software package ExomeDepth (v1.0.7)^39^ (494 samples) or using the VarSeq Copy number variation (CNV) software (184 samples) (Golden Helix, Montana, USA). The ExomeDepth uses read depth data to call CNVs. For each tested individual, the ExomeDepth algorithm builds the most appropriate reference set from the BAM files of a group of samples and ranks the CNV calls by their confidence level. The Varseq software generates a set of matched reference controls and the sample is compared to this set. A ratio and z-score is computed for each target region defined in the BED file. The z-scores measure the number of standard deviations that a sample’s coverage is from the mean coverage of the reference set. CNVs were validated either by MLPA, qPCR or chromosome microarray. MLPA was performed using kits P151 and P152 (*ABCA4*), P361 and P362 (*USH2A*), P328 (*EYS*) and P235 (*PRPF31*), following the manufacturer’s instructions (MRC-Holland, Amsterdam, the Netherlands). If no MLPA kit was available we performed qPCR using SYBR Power Green (Applied Biosystems, Foster City, CA, USA), 50 ng DNA and 12,5 pmol of each primer. Three primer sets were used to verify the aberrations and minimum two normal controls were included in the analysis. Real time PCR was run on a 7500 ABI SDS system (Applied Biosystems). Chromosome microarray was carried out using CytoScan HD array (Affymetrix, Santa Clara, CA, USA). Data analysis was performed using ChAS software (Affymetrix).

## Supplementary information

Supplementary info

Clinical diagnosis and genotypes of 487 individuals
