# Supplementary material for: Molecular genetic analysis using targeted NGS analysis of 677 individuals with retinal dystrophy
Source: Sci Rep. 2019 Feb 4;9:1219. doi: 10.1038/s41598-018-38007-2 (PMC6362094; doi:10.1038/s41598-018-38007-2)
Supplement: Supplementary file 1 — Supplementary info [file 41598_2018_38007_MOESM1_ESM.pdf]

Molecular genetic analysis using targeted NGS analysis of 677 individuals with retinal dystrophy

Cathrine Jespersgaard[1,+], Mingyan Fang[2,3,4+], Mette Bertelsen[1,5], Xiao Dang[2,3], Hanne Jensen[5] , Yulan Chen[2,3], Niels Bech[5] , Lanlan Dai[2,3], Thomas Rosenberg[5] , Jianguo Zhang[2,3], Lisbeth Birk Møller[1] , Zeynep Tümer[1,6], Karen Brøndum-Nielsen[1] and Karen Grønskov[1,*]

Table S1

**Diagnosis**	**Number of individuals**
**1. Generalized retinal dystrophy (non-syndromic)**	**486**
Retinitis pigmentosa	
Leber congenital amaurosis	
Cone-rod dystrophy	
Choroideremia	
Generalized choriocapillaris dystrophy (GCCD)	
Gyrate atrophy	
Unspecified generalized retinal and chorioretinal dystrophy	
**2. Isolated cone disease**	**24**
Cone dystrophy	
Achromatopsia	
Oligocone trichromacy	
**3. Macular dystrophy**	**118**
Best vitelliform macular dystrophy	
Adult –onset vitelliform macular dystrophy (including Pattern dystrophy)	
Stargardt disease (Stargardt macular dystrophy, fundus flavimaculatus)	
North Carolina macular dystrophy	
Macular dystrophy unspezified	
**4. Vitreoretinopathies**	**5**
Erosive vitreoretinopathies	
Enhanced S cone syndrome (ESCS) (incl Goldmann-Favre syndrome)	
Snowflake vitreoretinal degeneration	
Autosomal dominant vitreoretinochoroidopathy (ADVIRC)	
Wagner syndrome and erosive vitreoretinopathy	
Familial exudative vitreoretinopathies (FEVRs)incl Norrie disease	
X-linked juvenile retinoschises	
Vitreoretinopathy unspezified	
**5. Other non-syndromic retinal dystrophies**	**16**
Congenital stationary night blindness	
Bietti crystalline retinopathy	
Oculocutaneous albinism	
Åland eye disease	
Sorsby pseudoinflammatory fundus dystrophy	
Fundus albipunctatus	
**6. Syndromic retinal dystrophy**	**27**
Usher syndrome .	
Bardet Biedl syndrome	
Senior Løken Syndrome/Nephronophthisis (SLSN)	
Refsum disease	

Joubert syndrome	
Alagille syndrome (ALGS)	
Alstrom syndrome (ALMS)	
Neuronal ceroid lipofuscinosis (NCLs or CLNs)	
Primary ciliary dyskinesia (PCD)	
Unspezified syndrome	
**7. Retinal vascular dystrophies**	**1**

Table S2

Gene	OMIM gene	Inheritance	Phenotype	Number of mutations in HGMD (2018.2)	Function	NM-number	Gene ID
ABCA4	601691	AR	Stargardt disease; retinitis pigmentosa 19	1030	Visual cycle	NM_000350.2	NG_009073.1
ADAM9	602713	AR	Cone-rod dystrophy	8	Cell adhesion/structure	NM_003816.2	NG_016335.1
ADGRA3	*612303*	AR	Retinal dystrophy	4	Signal transduction (G-coupled 7 TM receptor)	NM_145290.3	NG_032963.1
ADGRV1	602851	AR	Usher syndrome 2C	184	Signal transduction (G-coupled 7 TM receptor)	NM_032119.3	NG_007083.2
AIPL1	604392	AR	Leber congenital amaurosis 4	70	Nuclear transport, protein trafficking, chaperone activity	NM_014336.4	NG_008474.1
ALMS1	606844	AR	Alstrom syndrome	291	Cilia function	NM_015120.4	NG_011690.1
ARL2BP	*615407*	AR	Retinitis pigmentosa +/- situs inversus	4	Transcription (cofactor)	NM_012106.3	NG_033905.1
ARL6	608845	AR	Bardet-Biedl syndrome 3	21	Cilia function	NM_177976.2	NG_008119.2
BBIP1	*613605*	AR	Bardet-Biedl syndrome 18	1	Cilia function	NM_001195306.1	NG_041778.1
BBS1	209901	AR	Bardet-Biedl syndrome 1	94	Cilia function	NM_024649.4	NG_009093.1
BBS10	610148	AR	Bardet-Biedl syndrome 10	92	Cilia function	NM_024685.3	NG_016357.1
BBS12	610683	AR	Bardet-Biedl syndrome 12	50	Cilia function	NM_152618.2	NG_021203.1
BBS2	606151	AR	Bardet-Biedl syndrome 2	81	Cilia function	NM_031885.3	NG_009312.1
BBS4	600374	AR	Bardet-Biedl syndrome 4	47	Cilia function	NM_033028.4	NG_009416.2
BBS5	603650	AR	Bardet-Biedl syndrome 5	25	Cilia function	NM_152384.2	NG_011567.1

BBS7	607590	AR	Bardet-Biedl syndrome 7	38	Cilia function	NM_176824.2	NG_009111.1
BBS9	607968	AR	Bardet-Biedl syndrome 9	41	Cilia function	NM_198428.2	NG_009306.2
BEST1 (AD)	607854	AD	Macular dystrophy, vitelliform 2	292	Ion channel (Ca2+)	NM_004183.3	NG_009033.1
BEST1 (AR)	607854	AR	Bestrophinopathy		Ion channel (Ca2+)	NM_004183.3	NG_009033.1
C1QTNF5	608752	AD	Retinal degeneration late onset	6	Cell adhesion/structure	NM_015645.4	NG_012235.1
C21ORF2	*603191*	AR	Early onset retinal dystrophy	17	Cilia function	NM_004928.2	NG_032952.1
PCARE	613425	AR	Retinitis pigmentosa 54	40	Cilia function	NM_001029883.2	NG_021427.1
C8ORF37	*614477*	AR	Bardet-Biedl syndrome 21; Cone-rod dystrophy 16; Retintis pigmentosa 64	12	Cilia function	NM_177965.3	NG_032804.1
CA4	114760	AD	Retinitis pigmentosa 17	8	Acid overload removal	NM_000717.3	NG_012050.2
CABP4	608965	AR	Cone-rod synaptic disorder, congenital non progressive	11	Synaptic function. Ca2+ influx regulator	NM_145200.3	NG_021211.1
CACNA1F	300110	XL	Åland eye disease; Night blindness congenital stationary	170	Ion channel (Ca2+)	NM_005183.3	NG_009095.2
CACNA2D4	608171	AR	Retinal cone dystrophy	4	Ion channel (Ca2+)	NM_172364.4	NG_012663.1
CDH23	605516	AR	Usher type 1D; deafness	288	Cell adhesion/structure	NM_022124.5	NG_008835.1
CDH3	114021	AR	Hypotrichosis congenital with juvenile macular dystrophy; Ectodermal	29	Cell adhesion/structure	NM_001793.4	NG_009096.1
CDHR1	609502	AR	Retintis pigmentosa 65; Cone-rod dystrophy 15	34	Cell adhesion/structure	NM_033100.3	NG_028034.1
CEP290	610142	AR	Leber congenital amaurosis 10; Meckel syndrome 4; Senior Loken syndrome 6;	262	Cilia function	NM_025114.3	NG_008417.1

CERKL	608381	AR	Retinitis pigmentosa 26	34	No information	NM_001030311.2	NG_021178.1
CHM	300390	XL	Choroideremia	273	Signal transduction	NM_000390.2	NG_009874.2
CLRN1	606397	AR	Usher syndrome type 3A; Retinitis pigmentosa 61	37	No information	NM_174878.2	NG_009168.1
CNGA1	123825	AR	Retinitis pigmentosa 49	27	Phototransduction	NM_000087.3	NG_009193.1
CNGB1	600724	AR	Retinitis pigmentosa 45	34	Phototransduction	NM_001297.4	NG_016351.1
CNGB3	605080	AR	Achromatopsia 3	114	Phototransduction	NM_019098.4	NG_016980.1
CNNM4	*607805*	AR	Jalili syndrome	23	No information	NM_020184.3	NG_016608.1
CRB1	604210	AR	Leber congenital amaurosis 8; Retinitis pigmentosa 12	299	Cell adhesion/structure	NM_201253.2	NG_008483.2
CRX	602225	AD	Cone-rod dystrophia; Leber congenital amaurosis 7	92	Transcription factor	NM_000554.4	NG_008605.1
DHDDS	608172	AR	Retintis pigmentosa 59	8	No information	NM_024887.3	NG_029786.1
ELOVL4	605512	AD	Stargardt disease 3	11	Cell adhesion/structure	NM_022726.3	NG_009108.1
EYS	612424	AR	Retintis pigmentosa 25	252	Cell adhesion/structure	NM_001142800.1	NG_023443.2
FAM161A	613596	AR	Retintis pigmentosa 28	17	Cilia function	NM_001201543.1	NG_028125.1
FLVCR1	609144	AR	Ataxia, posterior column, with retintis pigmentosa	14	Transport (heme)	NM_014053.3	NG_028131.1
FSCN2	607643	AD	Reported retinitis pigmentosa 30	1	No information	NM_001077182.2	NG_015964.1
GNAT1	139330	AD	Night blindness congenital stationary 3	7	Phototransduction	NM_144499.2	NG_009831.1

GPR179	614615	AR	Night blindness congenital stationary	14	Signal transduction (G-coupled 7 TM receptor)	NM_001004334.3	NG_032655.2
GRK1	180381	AR	Oguchi disease 2	16	Phototransduction	NM_002929.2	
GRM6	604096	AR	Night blindness congenital stationary 1B	35	Signal transduction (glutamate receptor)	NM_000843.3	NG_008105.1
GUCA1A	600364	AD	Cone dystrophy 3; Cone-rod dystrophy 14	20	Phototransduction	NM_000409.3	NG_009938.1
GUCA1B	602275	AD	Retinitis pigmentosa 48	4	Phototransduction	NM_002098.5	NG_016216.1
GUCY2D (AD)	600179	AD	Cone-rod dystrophy 6 (AD)	219	Phototransduction	NM_000180.3	NG_009092.1
GUCY2D (AR)	600179	AR	Leber congenital amaurosis 1 (AR);		Phototransduction	NM_000180.3	NG_009092.1
IDH3B	604526	AR	Retinitis pigmentosa 46	0	Citric acid cycle	NM_006899.3	NG_012149.1
IMPDH1	146690	AD	Retinitis pigmentosa 10; Leber congenital amaurosis 11	15	Cell growth	NM_000883.3	NG_009194.1
IMPG2	607056	AR	Retinitis pigmentosa 56; Macular dystrophy	34	Cell adhesion/structure	NM_016247.3	NG_028284.1
INPP5E	*613037*	AR	Joubert syndrome 1	46	Signal transduction	NM_019892.4	NG_016126.1
IQCB1	609237	AR	Senior Loken syndrome	39	Cilia function	NM_001023570.2	NG_015887.1
KCNV2	607604	AR	Retinal cone dystrophy	87	Ion channel (K+)	NM_133497.3	NG_012181.1
KLHL7	611119	AD	Retinitis pigmentosa 42	11	No information	NM_001031710.2	NG_016983.1
LCA5	*611408*	AR	Leber congenital amaurosis 5	47	Cilia function	NM_181714.3	NG_016011.1
LRAT	604863	AR	Leber congenital amaurosis 14	16	Visual cycle	NM_004744.4	NG_009110.1

LRIT3	*615004*	AR	Night blindness congenital stationary	5	Cell adhesion/structure	NM_198506.4	NG_033249.1
LZTFL1	*606568*	AR	Bardet-Biedl syndrome 17	3	Cilia function	NM_020347.3	NG_033917.1
MAK	*154235*	AR	Retinitis pigmentosa 62	16	Cilia function	NM_001242957.1	NG_030040.1
MERTK	604705	AR	Retinitis pigmentosa 38	58	Phagocytosis	NM_006343.2	NG_011607.1
MKKS	604896	AR	Bardet-Biedl syndrome 6; McKusick Kaufmann syndrome	55	Cilia function	NM_018848.3	NG_009109.1
MKS1	*609883*	AR	Bardet-Biedl syndrome13; Joubert syndrome; Meckel syndrome	46	Cilia function	NM_017777.3	NG_013032.1
MVK	*251170*	AR	Retinitis pigmentosa	169	Signal transduction	NM_000431.3	NG_007702.1
MYO7A	276903	AR	Usher syndrome 1B; isolated deafness	468	Cilia function	NM_000260.3	NG_009086.1
NR2E3 (AD)	604485	AD	Retinitis pigmentosa 37 (AD)	69	Transcription factor	NM_014249.3	NG_009113.2
NR2E3 (AR)	604485	AR	Enhanced S-cone syndrome (AR)		Transcription factor	NM_014249.3	NG_009113.2
NRL	162080	AD	Retinitis pigmentosa 27	23	Transcription factor	NM_006177.3	NG_011697.1
NYX	300278	XL	Night blindness congenital stationary	87	No information	NM_022567.2	NG_009112.1
OFD1	*300170*	XL	Joubert syndrome 10	152	Cilia function	NM_003611.2	NG_008872.1
PCDH15	605514	AR	Usher syndrome 1F; isolated deafness	101	Cell adhesion/structure	NM_033056.3	NG_009191.2
PDE6A	180071	AR	Retinitis pigmentosa 43	38	Phototransduction	NM_000440.2	NG_009102.1
PDE6B (AD)	180072	AD	NBSC (AD)	103	Phototransduction	NM_000283.3	NG_009839.1

PDE6B (AR)	180072	AR	Retinitis pigmentosa 40 (AR)		Phototransduction	NM_000283.3	NG_009839.1
PDE6C	600827	AR	Cone dystrophy 4	36	Phototransduction	NM_006204.3	NG_016752.1
PDE6G	180073	AR	Retinitis pigmentosa 57	2	Phototransduction	NM_002602.3	NG_009834.1
PDZD7	612971	?	Usher syndrome 2C	18	Cilia function	NM_001195263.1	NG_028030.1
PITPNM3	608921	AD	Cone-rod dystrophy 5	3	Signal transduction (phosphaditylinositol transfer	NM_031220.3	NG_016020.1
PRCD	610598	AR	Retinitis pigmentosa 36	6	No information	NM_001077620.2	NG_016702.1
PROM1	604365	AR	Cone-rod dystrophy 12; Macular dystrophy 2; Retinitis pigmentosa 41 (1	58	Cellular structure	NM_006017.2	NG_011696.1
PRPF3	607301	AD	Retinitis pigmentosa 18	6	Splicing factor	NM_004698.2	NG_008245.1
PRPF31	606419	AD	Retinitis pigmentosa 11	142	Splicing factor	NM_015629.3	NG_009759.1
PRPF6	*613979*	AD	Retinitis pigmentosa 60	4	Splicing factor	NM_012469.3	NG_029719.1
PRPF8	607300	AD	Retinitis pigmentosa 13	37	Splicing factor	NM_006445.3	NG_009118.1
PRPH2 (RDS)	179605	AD + DIGEN (+ROM1)	Retinitis pigmentosa 7; Leber congenital amaurosis 18; Macualr	161	Cell adhesion/structure	NM_000322.4	NG_009176.1
RAB28	*612994*	AR	Cone-rod dystrophy 18	4	Intracellular trafficing	NM_004249.3	NG_033891.1
RAX2	610362	AD	Cone-rod dystrophy 11	4	Transcription	NM_032753.3	NG_011565.1
RBP3	180290	AR	?Retinitis pigmentosa 66	8	Visual cycle	NM_002900.2	NG_029718.1
RBP4	180250	AR	Retinal dystrophy plus colobom; microphthalmia plus colobom	7	Visual cycle	NM_006744.3	NG_009104.1

RDH12	608830	AR	Leber congenital amaurosis 13	92	Visual cycle	NM_152443.2	NG_008321.1
RDH5	601617	AR	Fundus albipunctatus	48	Visual cycle	NM_002905.3	NG_008606.1
RGR	600342	AR	Retinitis pigmentosa 44	8	Signal transduction (G-coupled 7 TM receptor)	NM_001012720.1	NG_009106.1
RGS9	604067	AR	Bradyopsia	2	Phototransduction	NM_003835.3	NG_013021.1
RGS9BP	607814	AR	Bradyopsia	6	Phototransduction	NM_207391.2	NG_016751.1
RHO	180380	AD	Retinitis pigmentosa 4	198	Phototransduction	NM_000539.3	NG_009115.1
RIMS1	606629	AD	Cone-rod dystrophy 7	4	Exocytosis	NM_014989.5	NG_016209.1
RLBP1	180090	AR	Rod-cone dystrophy; retinitis puctata albescens	31	Visual cycle	NM_000326.4	NG_008116.1
ROM1	180721	DIGEN (+PRPH	Retinitis pigmentosa 7	11	Disc morphogenesis	NM_000327.3	NG_009845.1
RP1 (AD)	603937	AD	Retinitis pigmentosa 1	153	Cilia function	NM_006269.1	NG_009840.1
RP1 (AR)	603937	AR	Retinitis pigmentosa 1		Cilia function	NM_006269.1	NG_009840.1
RP1L1	608581	AR	Occult macular dystrophy	27	Cilia function	NM_178857.5	NG_028035.1
RP2	300757	XL	Retinitis pigmentosa 2	106	Signal transduction	NM_006915.2	NG_009107.1
RP9	607331	AD	?Retinitis pigmentosa 9	0	Splicing factor	NM_203288.1	NG_012968.1
RPE65	180069	AR	Leber congenital amaurosis 2; Retinitis pigmentosa 20	178	Visual cycle	NM_000329.2	NG_008472.1
RPGR	312610	XL	Cone-rod dystrophy 3; Retinitis pigmentosa 3	192	Cilia function	NM_000328.2	NG_009553.1

RPGRIP1	605446	AR	Leber congenital amaurosis 6; Cone-rod dystrophy 13	122	Cilia function	NM_020366.3	NG_008933.1
SAG	181031	AR	Oguchi disease; Retinitis pigmentosa 47	9	Phototransduction	NM_000541.4	NG_009116.1
SDCCAG8	*613524*	AR	Bardet-Biedl syndrome 16; Senior Loken syndrome	17	Cilia function	NM_006642.3	NG_027811.1
SEMA4A	607292	AR	Cone-rod dystrophy 10; Retinitis pigmentosa 35	7	Cell-cell signalling	NM_022367.3	NG_027683.1
SLC24A1	603617	AR	Night blindness congenital stationary	6	Phototransduction	NM_004727.2	NG_031968.2
SNRNP200	611664	AD	Retinitis pigmentosa 33	21	Splicing factor	NM_014014.4	NG_018973.1
SPATA7	609868	AR	Leber congenital amaurosis 3	36	Cilia function	NM_018418.4	NG_021183.1
TEAD1	*189967*	AD	Sveinsson chorioretinal atrophy	2	Transcription factor	NM_021961.5	NG_0021302.1
TIMP3	188826	AD	Sorsby fundus dystrophy	17	Matrix metalloproteinase	NM_000362.4	NG_009117.1
TOPORS	609507	AD	Retinitis pigmentosa 31	13	Cilia function	NM_005802.4	NG_017050.1
TRIM32	602290	AR	?Bardet-Biedl syndrome 11	15	E3 ubiquitin ligase	NM_012210.3	NG_011619.1
TRPM1	603576	AR	Night blindness congenital stationary	65	Ion channel (Ca2+)	NM_002420.5	NG_016453.2
TTC8	608132	AR	Bardet-Biedl syndrome 8	13	Cilia function	NM_198309.3	NG_008126.1
TULP1	302280	AR	Leber congenital amaurosis 15; Retinitis pigmentosa 14	62	Cilia function	NM_003322.4	NG_009077.1
USH1C	605242	AR	Usher syndrome 1C; isolated deafness	38	Scaffolding	NM_005709.3	NG_011883.1
USH1G	607696	AR	Usher asynrome 1G	28	Scaffolding	NM_173477.4	NG_007882.2

USH2A	608400	AR	Usher syndrome 2A; Retinitis pigmentosa 39	974	Cilia function	NM_206933.2	NG_009497.1
WHRN	607928	AR	Usher type 1D; Deafness 31	21	Cilia function	NM_015404.3	NG_016700.1
ZNF513	613598	AR	?Retinitis pigmentosa 58	1	Transcription factor (possible)	NM_144631.5	NG_028219.1

Table S3

**Target Capture Statistics**	**The average value for all samples**
Target region (bp)	2,168,860
Reads mapped to genome	15,845,300
Bases mapped to target regions (Mb)	1156.22
Reads mapped to target region	12,841,500
Reads mapped to flanking region	1,557,260
Mean depth of target region (X)	533.1
Mean depth of flanking regions(X)	122.24
Coverage of target region (%)	98.74
Fraction of target covered >=4X (%)	98.27
Fraction of target covered >=10X (%)	97.83
Fraction of target covered >=20X (%)	97.29

Table S5

No.	Type	Gene	Inheritance	Exons	Deletion/duplication	Second Variant
25	deletion	ABCA4	AR	ex 50	c.(6816+1_6817-1)_(*1_?)del#	c.1719G>A p.(Met573Ile)
39	deletion	AIPL1	AR	ex 1-2	c.(?_-1)_(276+1_277-1)del#	c.815G>C p.(Arg272Pro)
93	deletion	CNGB3	AR	ex 3	c.(211+1_212-1)_(338+1_339-1)del#<br>c.(211+1_212-1)_(338+1_339-1)del#	
399	duplication	CRX	AD	entire gene	c.(?_-1)_(*1_?)dup#	
121	deletion	EYS	AR	ex 13-22; 15-18;	c.(2023+1_2024-1)_(3443+1_3444-1)del#<br>c.(2023+1_2024-1)_(3443+1_3444-1)del#	
129	deletion	EYS	AR	ex 17-18	c.(2641+1_2642-1)_(2846+1_2847-1)del#	c.6714del p.(Ile2239Serfs*17)
403	deletion	EYS	AR	ex 30	c.(6078+1_6079-1)_(6191+1_6192-1)del	
126	duplication	EYS	AR	ex 14	c.(2137+1_2138-1)_(2259+1_2260-1)dup#	c.6714del p.(Ile2239Serfs*17)
122	duplication	EYS	AR	ex 30	c.(6078+1_6079-1)_(6191+1_6192-1)dup#<br>c.(6078+1_6079-1)_(6191+1_6192-1)dup#	
139	deletion	GRM6	AR	ex 7-9	c.(1354+1_1355-1)_(2436+1_2437-1)del#	c.1861C>T p.(Arg621*)
152	deletion	MERTK	AR	ex 1-7	c.(?_-1)_(1144+1_1145-1)del<br>c.(?_-1)_(1144+1_1145-1)del	
155	deletion	MERTK	AR	ex 1-7	c.(?_-1)_(1144+1_1145-1)del	c.757+1G>A p.(?)#
424	duplication	MERTK	AR	ex 2	c.(61+1_62-1)_(482+1_483-1)dup#	
189	deletion	PRPF31	AD	ex 2-14	c.(?_-1)_(*1_?)del#	
183	deletion	PRPF31	AD	ex 2-14	c.(?_-1)_(*1_?)del#	
194	deletion	PRPF31	AD	ex 2-3	c.(?_-1)_(238+1_239-1)del	
187	duplication	PRPF31	AD	ex 4-5	c.(238+1_239-1)_(420+1_421-1)dup#	
466	deletion	SAG	AR	ex 8-10	c.(512+1_513-1)_(806+1_807-1)del#	
271	deletion	USH2A	AR	ex 22-24	c.(4627+1_4628-1)_(4987+1_4988-1)del	c.12161G>T p.(Ser4054Ile)
297	duplication	USH2A	AR	ex 12-13-14	c.(1971+1_1972-1)_(2993+1_2994-1)dup#	c.10561T>C p.(Trp3521Arg)

Table S6

EYS

**HGVS c. (Clinically Relevant)**	**HGVS p. (Clinically Re**	**Chr:Pos**	**Ref/Alt**	**Identifier**	**Haplotype**
NM_001142800:c.8779T>C	p.Cys2927Arg	6:64431148	A/G	rs373203896	REF
NM_001142800:c.8071+84T>G		6:64472270	A/C	rs4710257	ALT
NM_001142800:c.7723+64T>A		6:64497934	A/T	rs72886371	REF
NM_001142800:c.7666A>T	p.Ser2556Cys	6:64498055	T/A	rs66462731	REF
NM_001142800:c.7228+1G>A		6:64574078	C/T		REF
NM_001142800:c.7228G>T	p.Ala2410Ser	6:64574079	C/A		REF
NM_001142800:c.6977G>A	p.Arg2326Gln	6:64694354	C/T	rs4710457	REF
NM_001142800:c.6835-64C>T		6:64694560	G/A	rs1482457	REF
NM_001142800:c.6835-124A>G		6:64694620	T/C	rs1482456	REF
NM_001142800:c.6834+61T>G		6:64708907	A/C	rs66502009	REF
NM_001142800:c.6725+130C>T		6:64776101	G/A	rs151263607	REF
NM_001142800:c.6714delT	p.Ile2239Serfs	6:64776242	A/-		REF
NM_001142800:c.6191+132G>A		6:65016731	C/T	rs9362694	ALT
NM_001142800:c.5928-35T>C		6:65098768	A/G	rs587278	ALT
NM_001142800:c.5244A>C	p.Leu1748Phe	6:65300516	T/G	rs57312007	REF
NM_001142800:c.4256T>C	p.Leu1419Ser	6:65301504	A/G	rs624851	REF
NM_001142800:c.3877+94delT		6:65302916	A/-	rs11331907	REF
NM_001142800:c.3444-5C>T		6:65336143	G/A	rs9445051	REF
NM_001142800:c.3443+160T>G		6:65523111	A/C	rs9345532	REF
NM_001142800:c.3243+219G>C		6:65531319	C/G	rs1552968	REF
NM_001142800:c.2847-24C>T		6:65596759	G/A	rs7743515	ALT
NM_001142800:c.2847-157A>G		6:65596892	T/C	rs58449017	REF
NM_001142800:c.2847-221C>T		6:65596956	G/A	rs59191409	REF
NM_001142800:c.2641+198A>C		6:65622179	T/G	rs10046141	ALT
NM_001142800:c.2555T>C	p.Leu852Pro	6:65622463	A/G	rs9294631	ALT
NM_001142800:c.2382-26C>G		6:65622662	G/C	rs9445437	ALT
NM_001142800:c.2259+164delT		6:65707311	A/-	rs376045129	REF
NM_001142800:c.2138-197dupT		6:65707793	-/A	rs200128743	REF
NM_001142800:c.2137+114C>T		6:65767393	G/A	rs10455568	ALT
NM_001142800:c.2024-14_2024-13insT		6:65767634	-/A	rs142590049	REF
NM_001142800:c.2024-14C>T		6:65767634	G/A	rs45628235	REF
NM_001142800:c.2023+146delA		6:66005610	T/-	rs34666704	ALT
NM_001142800:c.2023+15dupT		6:66005741	-/A	rs374955689	REF
NM_001142800:c.2023+15delT		6:66005741	A/-	rs374955689	ALT
NM_001142800:c.1922A>T	p.Glu641Val	6:66005857	T/A	rs17411795	REF
NM_001142800:c.1891G>A	p.Gly631Ser	6:66005888	C/T	rs9342464	REF
NM_001142800:c.1809C>T	p.Val603=	6:66005970	G/A	rs9345601	REF
NM_001142800:c.1767-136T>G		6:66006148	A/C	rs9342465	REF
NM_001142800:c.1766+6309T>C		6:66038564	A/G	rs61283975	ALT
NM_001142800:c.1766+6255C>T		6:66038618	G/A	rs1827476	ALT
NM_001142800:c.1766+5997G>A		6:66038876	C/T	rs35176562	REF
NM_001142800:c.1766+5933T>A		6:66038940	A/T	rs114665982	ALT
NM_001142800:c.1766+5415C>T		6:66039458	G/A	rs9354227	ALT
NM_001142800:c.1766+5272C>T		6:66039601	G/A	rs9354228	REF
NM_001142800:c.1766+4413G>T		6:66040460	C/A	rs1502967	ALT
NM_001142800:c.1766+4320G>A		6:66040553	C/T	rs7755788	ALT
NM_001142800:c.1766+3945A>G		6:66040928	T/C	rs71572524	REF

Table S6

NM_001142800:c.1766+3639T>C		6:66041234	A/G	rs4710516	REF
NM_001142800:c.1766+3630G>A		6:66041243	C/T	rs34794632	REF
NM_001142800:c.1766+3555C>A		6:66041318	G/T	rs4710517	REF
NM_001142800:c.1766+2878C>T		6:66041995	G/A	rs12212303	ALT
NM_001142800:c.1766+2642A>C		6:66042231	T/G	rs12207746	ALT
NM_001142800:c.1766+1153T>G		6:66043720	A/C	rs9360123	REF
NM_001142800:c.1766+1065dupA		6:66043808	-/T	rs148046154	ALT
NM_001142800:c.1766+61A>G		6:66044812	T/C	rs76148513	ALT
NM_001142800:c.1600-38G>A		6:66045077	C/T	rs1502965	ALT
NM_001142800:c.1600-79A>G		6:66045118	T/C	rs1502964	ALT
NM_001142800:c.1599+96A>C		6:66053835	T/G	rs1502963	ALT
NM_001142800:c.1300-3C>T		6:66063513	G/A	rs1936439	ALT
NM_001142800:c.1146T>C	p.Asn382=	6:66112409	A/G	rs974110	REF
NM_001142800:c.862+87T>C		6:66200400	A/G	rs4710522	REF
NM_001142800:c.748+225G>A		6:66204331	C/T	rs35009856	REF
NM_001142800:c.359C>T	p.Thr120Met	6:66204945	G/A	rs12193967	ALT
NM_001142800:c.232delT	p.Cys78Alafs	6:66205072	A/-		ALT
NM_001142800:c.-198+83T>A		6:66205669	A/T	rs13217899	REF
NM_001142800:c.-332-1124A>G		6:66207010	T/C	rs12193142	ALT
NM_001142800:c.-332-1332T>C		6:66207218	A/G	rs10944813	ALT
NM_001142800:c.-332-2668delT		6:66208554	A/-	rs201814171	ALT
NM_001142800:c.-332-2745A>G		6:66208631	T/C	rs4710523	REF
NM_001142800:c.-332-2748A>G		6:66208634	T/C	rs9345642	ALT
NM_001142800:c.-332-4926A>G		6:66210812	T/C	rs9360133	ALT
NM_001142800:c.-332-5141A>C		6:66211027	T/G	rs13193142	REF
NM_001142800:c.-332-5493G>A		6:66211379	C/T	rs3904332	ALT
NM_001142800:c.-332-7111T>A		6:66212997	A/T	rs34706159	REF
NM_001142800:c.-332-7760C>T		6:66213646	G/A	rs4710292	ALT
NM_001142800:c.-332-8122G>A		6:66214008	C/T	rs4710524	ALT
NM_001142800:c.-332-8216A>T		6:66214102	T/A	rs4710293	REF
NM_001142800:c.-332-8220delT		6:66214106	A/-	rs77754448	ALT
NM_001142800:c.-332-8667T>A		6:66214553	A/T	rs9345643	ALT
NM_001142800:c.-332-8831C>T		6:66214717	G/A	rs9363380	ALT
NM_001142800:c.-332-9451C>G		6:66215337	G/C	rs1576638	ALT
NM_001142800:c.-332-9663C>T		6:66215549	G/A	rs7449561	ALT
NM_001142800:c.-332-10150C>G		6:66216036	G/C	rs9351508	ALT
NM_001142800:c.-332-10655dupA		6:66216541	-/T	rs35607551	ALT
NM_001142800:c.-332-11104A>G		6:66216990	T/C	rs9360134	ALT
NM_001142800:c.-332-11649T>C		6:66217535	A/G	rs1576639	ALT
NM_001142800:c.-332-11749G>C		6:66217635	C/G	rs9345644	ALT
NM_001142800:c.-332-12670delT		6:66218556	A/-	rs35302193	0/1
NM_001142800:c.-332-12688C>T		6:66218574	G/A		REF
NM_001142800:c.-332-12692C>T		6:66218578	G/A		REF
NM_001142800:c.-332-13032T>G		6:66218918	A/C	rs9363381	ALT
NM_001142800:c.-332-13085G>A		6:66218971	C/T	rs6924402	ALT
NM_001142800:c.-332-13209C>G		6:66219095	G/C	rs7759568	ALT
NM_001142800:c.-332-13935G>A		6:66219821	C/T	rs60010253	REF
NM_001142800:c.-332-13954G>T		6:66219840	C/A	rs9363382	ALT

Table S6

NM_001142800:c.-332-14016dupA	6:66219902	-/T	rs112450408	REF
NM_001142800:c.-332-14352T>G	6:66220238	A/C		REF
NM_001142800:c.-332-14359dupA	6:66220245	-/T		REF
NM_001142800:c.-332-14399C>G	6:66220285	G/C		REF
NM_001142800:c.-332-15373A>T	6:66221259	T/A	rs6931758	0/1
NM_001142800:c.-332-16016C>T	6:66221902	G/A		REF
NM_001142800:c.-332-16093T>G	6:66221979	A/C	rs2882615	ALT
NM_001142800:c.-332-19729T>C	6:66225615	A/G		REF
NM_001142800:c.-332-19754G>T	6:66225640	C/A	rs12199163	ALT
NM_001142800:c.-332-19758G>A	6:66225644	C/T		REF
NM_001142800:c.-332-19960T>C	6:66225846	A/G	rs138927014	REF
NM_001142800:c.-332-20197C>T	6:66226083	G/A	rs11759352	REF
NM_001142800:c.-332-20453T>C	6:66226339	A/G	rs7751898	REF
NM_001142800:c.-332-20695T>G	6:66226581	A/C	rs7752247	REF
NM_001142800:c.-332-20996A>T	6:66226882	T/A	rs13208352	REF
NM_001142800:c.-332-21349dupT	6:66227235	-/A	rs71002314	1/.
NM_001142800:c.-332-21366A>G	6:66227252	T/C	rs3846800	ALT
NM_001142800:c.-332-23352C>A	6:66229238	G/T		REF
NM_001142800:c.-332-23600A>T	6:66229486	T/A	rs9453315	REF
NM_001142800:c.-332-23752A>G	6:66229638	T/C	rs9445551	REF
NM_001142800:c.-332-23899dupA	6:66229785	-/T		0/1
NM_001142800:c.-332-23899delA	6:66229785	T/-	rs34941445	REF
NM_001142800:c.-332-23901A>T	6:66229787	T/A	rs34303504	REF
NM_001142800:c.-332-24340C>T	6:66230226	G/A	rs9342483	REF
NM_001142800:c.-332-25178T>C	6:66231064	A/G	rs7749840	ALT
NM_001142800:c.-332-26229A>G	6:66232115	T/C	rs4710294	REF
NM_001142800:c.-332-26634T>G	6:66232520	A/C	rs1884374	REF
NM_001142800:c.-332-26702C>T	6:66232588	G/A	rs1884373	REF
NM_001142800:c.-332-26712A>C	6:66232598	T/G	rs1884372	REF
NM_001142800:c.-332-26750A>T	6:66232636	T/A	rs13208103	REF
NM_001142800:c.-332-27007G>A	6:66232893	C/T	rs9445553	REF
NM_001142800:c.-332-27662C>G	6:66233548	G/C	rs9445554	REF
NM_001142800:c.-332-27708C>A	6:66233594	G/T	rs9354259	REF
NM_001142800:c.-332-28171T>A	6:66234057	A/T	rs35348004	REF
NM_001142800:c.-332-28851T>A	6:66234737	A/T	rs9363384	REF
NM_001142800:c.-332-28935C>T	6:66234821	G/A	rs9354260	REF
NM_001142800:c.-332-29063dupA	6:66234949	-/T	rs200412074	0/1
NM_001142800:c.-332-29329G>C	6:66235215	C/G	rs9363385	REF
NM_001142800:c.-332-29421G>A	6:66235307	C/T	rs9453319	REF
NM_001142800:c.-332-29593A>C	6:66235479	T/G	rs9354261	ALT
NM_001142800:c.-332-30087G>C	6:66235973	C/G	rs4710295	REF
NM_001142800:c.-332-30174G>A	6:66236060	C/T	rs4710296	REF
NM_001142800:c.-332-30224A>T	6:66236110	T/A	rs7742748	REF
NM_001142800:c.-332-30594G>A	6:66236480	C/T	rs1929326	REF

Table S6

NM_001142800:c.-332-30672C>T	6:66236558	G/A	rs10944814	ALT
NM_001142800:c.-332-30674C>A	6:66236560	G/T	rs1929327	REF
NM_001142800:c.-332-31374C>G	6:66237260	G/C	rs1929328	REF
NM_001142800:c.-332-31423T>C	6:66237309	A/G	rs1034137	ALT
NM_001142800:c.-332-31532A>G	6:66237418	T/C	rs9345645	ALT
NM_001142800:c.-332-31951C>T	6:66237837	G/A	rs35052047	REF
NM_001142800:c.-332-32049G>A	6:66237935	C/T	rs2024792	ALT
NM_001142800:c.-332-32434G>A	6:66238320	C/T	rs7768271	REF
NM_001142800:c.-332-33086G>A	6:66238972	C/T	rs10484938	REF
NM_001142800:c.-332-33147dupT	6:66239033	-/A		REF
NM_001142800:c.-332-34141T>G	6:66240027	A/C	rs9363386	ALT
NM_001142800:c.-332-34510T>A	6:66240396	A/T	rs10484937	REF
NM_001142800:c.-332-34831G>A	6:66240717	C/T	rs9453321	REF
NM_001142800:c.-332-34976A>G	6:66240862	T/C	rs7740699	ALT
NM_001142800:c.-332-35001A>G	6:66240887	T/C	rs9354262	REF
NM_001142800:c.-332-35300T>A	6:66241186	A/T	rs7775434	REF
NM_001142800:c.-332-35696G>C	6:66241582	C/G	rs7756463	REF
NM_001142800:c.-332-35718T>C	6:66241604	A/G	rs7776123	REF
NM_001142800:c.-332-35922C>G	6:66241808	G/C	rs11963174	REF
NM_001142800:c.-332-36743T>C	6:66242629	A/G	rs66608147	REF
NM_001142800:c.-332-36992A>G	6:66242878	T/C	rs10484936	REF
NM_001142800:c.-332-36996G>A	6:66242882	C/T	rs2351260	ALT
NM_001142800:c.-332-37235G>A	6:66243121	C/T		REF
NM_001142800:c.-332-37239A>C	6:66243125	T/G		REF
NM_001142800:c.-332-37430G>A	6:66243316	C/T	rs62407706	REF
NM_001142800:c.-332-37483G>C	6:66243369	C/G	rs115897734	REF
NM_001142800:c.-332-37524C>G	6:66243410	G/C		REF
NM_001142800:c.-332-37535A>G	6:66243421	T/C		REF
NM_001142800:c.-332-37981dupA	6:66243867	-/T		REF
NM_001142800:c.-332-38237G>A	6:66244123	C/T	rs1831476	REF
NM_001142800:c.-332-40332dupA	6:66246218	-/T		0/1
NM_001142800:c.-332-40332delA	6:66246218	T/-	rs71864647	REF
NM_001142800:c.-332-40724G>A	6:66246610	C/T	rs1929330	REF
NM_001142800:c.-332-40939C>T	6:66246825	G/A	rs7744348	REF
NM_001142800:c.-332-41153C>T	6:66247039	G/A		REF
NM_001142800:c.-332-41164G>A	6:66247050	C/T		REF
NM_001142800:c.-332-41165C>T	6:66247051	G/A	rs9294641	REF
NM_001142800:c.-332-41643T>G	6:66247529	A/C	rs2210338	ALT
NM_001142800:c.-332-41717A>G	6:66247603	T/C	rs9294642	REF
NM_001142800:c.-332-42339A>C	6:66248225	T/G	rs2039311	ALT
NM_001142800:c.-332-42401T>C	6:66248287	A/G	rs9363387	REF
NM_001142800:c.-332-42464C>G	6:66248350	G/C	rs9354263	REF
NM_001142800:c.-332-42538A>G	6:66248424	T/C	rs1411850	REF
NM_001142800:c.-332-42573A>G	6:66248459	T/C	rs12175029	ALT
NM_001142800:c.-332-42984T>A	6:66248870	A/T	rs9351509	ALT
NM_001142800:c.-332-43299G>A	6:66249185	C/T	rs115439766	ALT

Table S6

NM_001142800:c.-332-43500C>T	6:66249386	G/A	rs6923660	REF
NM_001142800:c.-332-43614A>G	6:66249500	T/C	rs16896814	REF
NM_001142800:c.-332-43724A>G	6:66249610	T/C	rs6909236	ALT
NM_001142800:c.-332-43938G>T	6:66249824	C/A	rs1331200	REF
NM_001142800:c.-332-44011C>T	6:66249897	G/A	rs1331202	ALT
NM_001142800:c.-332-44857C>T	6:66250743	G/A	rs10944815	ALT
NM_001142800:c.-332-44966G>A	6:66250852	C/T	rs6904086	ALT
NM_001142800:c.-332-45071A>G	6:66250957	T/C	rs2039312	ALT
NM_001142800:c.-332-45095dupA	6:66250981	-/T	rs35370331	ALT
NM_001142800:c.-332-45202T>C	6:66251088	A/G	rs1543666	ALT
NM_001142800:c.-332-45415delA	6:66251301	T/-	rs5876967	ALT
NM_001142800:c.-332-45666delT	6:66251552	A/-	rs71553518	REF
NM_001142800:c.-332-45724A>C	6:66251610	T/G	rs1556391	REF
NM_001142800:c.-332-45743C>T	6:66251629	G/A	rs1556392	ALT
NM_001142800:c.-332-45871C>T	6:66251757	G/A	rs9342484	ALT
NM_001142800:c.-332-46044A>G	6:66251930	T/C	rs1556394	REF
NM_001142800:c.-332-46099A>G	6:66251985	T/C	rs16896824	REF
NM_001142800:c.-332-46114A>G	6:66252000	T/C	rs9363388	REF
NM_001142800:c.-332-46125T>A	6:66252011	A/T	rs9363389	REF
NM_001142800:c.-332-46134T>A	6:66252020	A/T	rs9345647	ALT
NM_001142800:c.-332-46875A>G	6:66252761	T/C	rs16896825	REF
NM_001142800:c.-332-47274T>C	6:66253160	A/G	rs6939055	ALT
NM_001142800:c.-332-47390T>C	6:66253276	A/G	rs6939243	ALT
NM_001142800:c.-332-47995G>A	6:66253881	C/T	rs6922236	ALT
NM_001142800:c.-332-48038T>A	6:66253924	A/T	rs5008809	0/1
NM_001142800:c.-332-48040T>A	6:66253926	A/T	rs5008810	0/1
NM_001142800:c.-332-48198A>G	6:66254084	T/C	rs1411856	ALT
NM_001142800:c.-332-48281G>A	6:66254167	C/T	rs1411857	ALT
NM_001142800:c.-332-48414A>G	6:66254300	T/C	rs1411858	ALT
NM_001142800:c.-332-48421G>A	6:66254307	C/T	rs1411859	ALT
NM_001142800:c.-332-48440A>G	6:66254326	T/C	rs5001652	REF
NM_001142800:c.-332-48515G>A	6:66254401	C/T	rs4501413	ALT
NM_001142800:c.-332-48974T>C	6:66254860	A/G	rs1331208	ALT
NM_001142800:c.-332-49181G>A	6:66255067	C/T	rs2351261	ALT
NM_001142800:c.-332-49526T>C	6:66255412	A/G	rs1331209	ALT
NM_001142800:c.-332-49676G>A	6:66255562	C/T	rs1331210	ALT
NM_001142800:c.-332-49701G>T	6:66255587	C/A	rs1331211	ALT
NM_001142800:c.-332-49902G>T	6:66255788	C/A	rs972709	ALT
NM_001142800:c.-332-50031C>A	6:66255917	G/T	rs972710	ALT
NM_001142800:c.-332-50066T>C	6:66255952	A/G	rs972711	ALT
NM_001142800:c.-332-50193delA	6:66256079	T/-	rs5876969	REF
NM_001142800:c.-332-50267G>A	6:66256153	C/T	rs972712	ALT
NM_001142800:c.-332-50329delA	6:66256215	T/-	rs111400363	ALT
NM_001142800:c.-332-50537G>T	6:66256423	C/A	rs9345648	REF
NM_001142800:c.-332-50581dupA	6:66256467	-/T	rs11396973	ALT

Table S6

NM_001142800:c.-332-50634T>C	6:66256520	A/G	rs6914700	ALT
NM_001142800:c.-332-50751A>G	6:66256637	T/C	rs6919610	REF
NM_001142800:c.-332-50864C>T	6:66256750	G/A	rs34586455	REF
NM_001142800:c.-332-51310T>C	6:66257196	A/G	rs9363391	ALT
NM_001142800:c.-332-52016T>A	6:66257902	A/T	rs1411839	ALT
NM_001142800:c.-332-52081C>T	6:66257967	G/A	rs1411840	REF
NM_001142800:c.-332-52214A>G	6:66258100	T/C	rs2351262	ALT
NM_001142800:c.-332-52293T>G	6:66258179	A/C	rs1411841	ALT
NM_001142800:c.-332-52347C>T	6:66258233	G/A	rs1929331	ALT
NM_001142800:c.-332-52717T>A	6:66258603	A/T	rs1929332	ALT
NM_001142800:c.-332-52736G>C	6:66258622	C/G	rs1929333	ALT
NM_001142800:c.-332-52765G>A	6:66258651	C/T	rs1929334	ALT
NM_001142800:c.-332-52892A>G	6:66258778	T/C	rs1331197	ALT
NM_001142800:c.-332-53065G>T	6:66258951	C/A	rs9363392	ALT
NM_001142800:c.-332-53332C>A	6:66259218	G/T	rs76838586	REF
NM_001142800:c.-332-53472A>G	6:66259358	T/C	rs6937452	ALT
NM_001142800:c.-332-53524A>C	6:66259410	T/G	rs6937478	ALT
NM_001142800:c.-332-53535C>T	6:66259421	G/A	rs6455046	ALT
NM_001142800:c.-332-53622T>C	6:66259508	A/G	rs6932966	ALT
NM_001142800:c.-332-53651C>T	6:66259537	G/A	rs6909711	ALT
NM_001142800:c.-332-53660T>C	6:66259546	A/G	rs6455047	REF
NM_001142800:c.-332-53827C>T	6:66259713	G/A	rs9354264	ALT
NM_001142800:c.-332-53945A>G	6:66259831	T/C	rs2351263	ALT
NM_001142800:c.-332-53995G>A	6:66259881	C/T	rs9345649	ALT
NM_001142800:c.-332-54005A>G	6:66259891	T/C	rs2351264	ALT
NM_001142800:c.-332-54305dupA	6:66260191	-/T		REF
NM_001142800:c.-332-54343dupA	6:66260229	-/T		REF
NM_001142800:c.-332-54347dupA	6:66260233	-/T		REF
NM_001142800:c.-332-54351A>T	6:66260237	T/A		REF
NM_001142800:c.-332-54353A>T	6:66260239	T/A		REF
NM_001142800:c.-332-56170C>T	6:66262056	G/A	rs4507566	ALT
NM_001142800:c.-332-56229T>G	6:66262115	A/C	rs4460199	ALT
NM_001142800:c.-332-56346C>T	6:66262232	G/A	rs4524589	ALT
NM_001142800:c.-332-56445A>C	6:66262331	T/G	rs9453326	ALT
NM_001142800:c.-332-56452C>T	6:66262338	G/A	rs9363393	ALT
NM_001142800:c.-332-56520A>G	6:66262406	T/C	rs4506027	ALT
NM_001142800:c.-332-56615A>T	6:66262501	T/A	rs9342485	ALT
NM_001142800:c.-332-56720T>G	6:66262606	A/C	rs9360135	ALT
NM_001142800:c.-332-56809A>G	6:66262695	T/C	rs9345650	ALT
NM_001142800:c.-332-56834C>T	6:66262720	G/A	rs3908650	ALT
NM_001142800:c.-332-56933A>G	6:66262819	T/C	rs3908651	ALT
NM_001142800:c.-332-56935C>T	6:66262821	G/A	rs9363394	ALT
NM_001142800:c.-332-57162G>A	6:66263048	C/T	rs3908652	ALT
NM_001142800:c.-332-57335T>C	6:66263221	A/G	rs7452712	ALT
NM_001142800:c.-332-57368T>G	6:66263254	A/C	rs9354265	ALT
NM_001142800:c.-332-57696A>T	6:66263582	T/A	rs3846802	ALT
NM_001142800:c.-332-57768A>G	6:66263654	T/C	rs3857534	ALT

Table S6

NM_001142800:c.-332-57852G>T	6:66263738	C/A	rs3857535	REF
NM_001142800:c.-332-58098G>A	6:66263984	C/T	rs2150618	ALT
NM_001142800:c.-332-58293G>A	6:66264179	C/T	rs2150619	ALT
NM_001142800:c.-332-58500T>A	6:66264386	A/T	rs2351265	ALT
NM_001142800:c.-332-58552A>C	6:66264438	T/G	rs6935790	ALT
NM_001142800:c.-332-58621T>C	6:66264507	A/G	rs1411847	ALT
NM_001142800:c.-332-58849A>G	6:66264735	T/C	rs1411848	ALT
NM_001142800:c.-332-58869C>T	6:66264755	G/A	rs1411849	ALT
NM_001142800:c.-332-59017delT	6:66264903	A/-	rs5876972	ALT
NM_001142800:c.-332-59045T>C	6:66264931	A/G	rs1929335	ALT
NM_001142800:c.-332-59217A>G	6:66265103	T/C	rs1929336	ALT
NM_001142800:c.-332-59938T>G	6:66265824	A/C	rs1999363	ALT
NM_001142800:c.-332-60418T>C	6:66266304	A/G	rs2351266	ALT
NM_001142800:c.-332-60657A>G	6:66266543	T/C	rs7743334	REF
NM_001142800:c.-332-60690G>A	6:66266576	C/T	rs1929337	ALT
NM_001142800:c.-332-61647C>T	6:66267533	G/A	rs6926034	ALT
NM_001142800:c.-332-61961G>C	6:66267847	C/G	rs6932247	ALT
NM_001142800:c.-332-62068G>A	6:66267954	C/T	rs7767609	REF
NM_001142800:c.-332-62297G>A	6:66268183	C/T	rs9360136	ALT
NM_001142800:c.-332-62310T>C	6:66268196	A/G	rs1411852	ALT
NM_001142800:c.-332-62543C>T	6:66268429	G/A	rs1411853	REF
NM_001142800:c.-332-62874C>T	6:66268760	G/A	rs1473778	ALT
NM_001142800:c.-332-62900A>T	6:66268786	T/A	rs1473779	ALT
NM_001142800:c.-332-63513T>C	6:66269399	A/G	rs1331205	REF
NM_001142800:c.-332-63845T>A	6:66269731	A/T	rs9345651	ALT
NM_001142800:c.-332-64004C>A	6:66269890	G/T	rs4710526	ALT
NM_001142800:c.-332-64078A>G	6:66269964	T/C	rs1473780	ALT
NM_001142800:c.-332-64818G>A	6:66270704	C/T	rs9363395	ALT
NM_001142800:c.-332-65097C>T	6:66270983	G/A	rs9345652	REF
NM_001142800:c.-332-65268T>G	6:66271154	A/C	rs1411855	ALT
NM_001142800:c.-332-65514G>T	6:66271400	C/A	rs2225457	ALT
NM_001142800:c.-332-65551G>A	6:66271437	C/T	rs13204681	REF
NM_001142800:c.-332-65557G>A	6:66271443	C/T	rs9363396	ALT
NM_001142800:c.-332-66014C>T	6:66271900	G/A	rs7349900	ALT
NM_001142800:c.-332-67582dupA	6:66273468	-/T	rs201551142	0/1
NM_001142800:c.-332-67590T>A	6:66273476	A/T	rs1331206	ALT
NM_001142800:c.-332-67724A>G	6:66273610	T/C	rs1331207	ALT
NM_001142800:c.-332-67918A>C	6:66273804	T/G		REF
NM_001142800:c.-332-67921C>T	6:66273807	G/A		REF
NM_001142800:c.-332-67973T>C	6:66273859	A/G		REF
NM_001142800:c.-332-67976T>A	6:66273862	A/T		REF
NM_001142800:c.-332-69675C>A	6:66275561	G/T	rs55792659	REF
NM_001142800:c.-332-69906A>C	6:66275792	T/G	rs12198506	ALT
NM_001142800:c.-332-69915G>A	6:66275801	C/T	rs71572536	REF
NM_001142800:c.-332-70103dupT	6:66275989	-/A	rs368929865	0/1
NM_001142800:c.-332-70135C>G	6:66276021	G/C	rs12204695	ALT
NM_001142800:c.-332-70347C>T	6:66276233	G/A	rs12204856	ALT
NM_001142800:c.-332-70468G>T	6:66276354	C/A	rs12191952	ALT

Table S6

NM_001142800:c.-332-70670A>T	6:66276556	T/A	rs62407713	REF
NM_001142800:c.-332-70756T>C	6:66276642	A/G	rs4314478	ALT
NM_001142800:c.-332-71287G>A	6:66277173	C/T	rs4235971	ALT
NM_001142800:c.-332-71530C>T	6:66277416	G/A	rs9342486	REF
NM_001142800:c.-332-71742C>G	6:66277628	G/C	rs34412399	REF
NM_001142800:c.-333+71756C>A	6:66277915	G/T	rs7753548	ALT
NM_001142800:c.-333+71469delA	6:66278202	T/-	rs61492530	./1
NM_001142800:c.-333+71096C>T	6:66278575	G/A	rs13203580	REF
NM_001142800:c.-333+70597G>T	6:66279074	C/A	rs9345653	ALT
NM_001142800:c.-333+70136G>C	6:66279535	C/G	rs9345654	ALT
NM_001142800:c.-333+69967C>G	6:66279704	G/C	rs9363398	REF
NM_001142800:c.-333+69650dupT	6:66280021	-/A		REF
NM_001142800:c.-333+69646delA	6:66280025	T/-	rs9363399,rs67[illegible]	0/1
NM_001142800:c.-333+69646A>T	6:66280025	T/A	rs9363399,rs67[illegible]	0/1
NM_001142800:c.-333+69606T>C	6:66280065	A/G	rs66812553	ALT
NM_001142800:c.-333+69487T>C	6:66280184	A/G	rs9360138	ALT
NM_001142800:c.-333+68564G>A	6:66281107	C/T	rs34268718	REF
NM_001142800:c.-333+68363G>C	6:66281308	C/G	rs6455048	REF
NM_001142800:c.-333+67794T>C	6:66281877	A/G	rs12190494	ALT
NM_001142800:c.-333+67447G>A	6:66282224	C/T	rs6650991	ALT
NM_001142800:c.-333+67298G>A	6:66282373	C/T	rs1331203	ALT
NM_001142800:c.-333+67285C>G	6:66282386	G/C	rs1331204	ALT
NM_001142800:c.-333+65971delT	6:66283700	A/-	rs61328716	REF
NM_001142800:c.-333+65871G>A	6:66283800	C/T	rs9351510	REF
NM_001142800:c.-333+65485C>T	6:66284186	G/A	rs9360139	ALT
NM_001142800:c.-333+65425C>G	6:66284246	G/C	rs9363400	ALT
NM_001142800:c.-333+65421T>A	6:66284250	A/T	rs1590965	ALT
NM_001142800:c.-333+65029A>G	6:66284642	T/C	rs76704490	REF
NM_001142800:c.-333+65018C>G	6:66284653	G/C	rs1590966	ALT
NM_001142800:c.-333+64865T>C	6:66284806	A/G	rs13328254	REF
NM_001142800:c.-333+64863A>G	6:66284808	T/C	rs1590967	REF
NM_001142800:c.-333+63980T>C	6:66285691	A/G	rs9294643	ALT
NM_001142800:c.-333+63713G>A	6:66285958	C/T	rs9351511	ALT
NM_001142800:c.-333+63615G>C	6:66286056	C/G	rs354346	ALT
NM_001142800:c.-333+63216A>G	6:66286455	T/C	rs354347	ALT
NM_001142800:c.-333+63009G>A	6:66286662	C/T	rs354348	ALT
NM_001142800:c.-333+62748T>A	6:66286923	A/T	rs10944816	ALT
NM_001142800:c.-333+62306G>T	6:66287365	C/A	rs354349	ALT
NM_001142800:c.-333+62129A>G	6:66287542	T/C	rs354350	ALT
NM_001142800:c.-333+62028_-333+62029insT	6:66287643	-/A	rs147331700	REF
NM_001142800:c.-333+62028A>T	6:66287643	T/A	rs61475327	REF
NM_001142800:c.-333+62027dupT	6:66287644	-/A	rs61475327	1/.
NM_001142800:c.-333+61711T>G	6:66287960	A/C	rs2776773	ALT
NM_001142800:c.-333+61622A>G	6:66288049	T/C	rs354351	ALT
NM_001142800:c.-333+60997A>G	6:66288674	T/C	rs354352	ALT

Table S6

NM_001142800:c.-333+60488A>G	6:66289183	T/C	rs1929338	REF
NM_001142800:c.-333+58923T>C	6:66290748	A/G	rs186438553	REF
NM_001142800:c.-333+58528T>C	6:66291143	A/G		REF
NM_001142800:c.-333+57657T>C	6:66292014	A/G	rs354354	ALT
NM_001142800:c.-333+55810dupT	6:66293861	-/A		REF
NM_001142800:c.-333+55810delT	6:66293861	A/-	rs61362090	REF
NM_001142800:c.-333+54846A>G	6:66294825	T/C	rs354358	ALT
NM_001142800:c.-333+53899C>T	6:66295772	G/A	rs374738	ALT
NM_001142800:c.-333+53641C>T	6:66296030	G/A	rs35936441	REF
NM_001142800:c.-333+53476G>C	6:66296195	C/G	rs354359	ALT
NM_001142800:c.-333+52809T>C	6:66296862	A/G	rs188298223	REF
NM_001142800:c.-333+51495A>G	6:66298176	T/C	rs354361	ALT
NM_001142800:c.-333+51358G>A	6:66298313	C/T	rs6902867	REF
NM_001142800:c.-333+51214delA	6:66298457	T/-	rs35933552	ALT
NM_001142800:c.-333+49538T>C	6:66300133	A/G	rs13194431	REF
NM_001142800:c.-333+49184A>G	6:66300487	T/C	rs34018715	REF
NM_001142800:c.-333+48967dupA	6:66300704	-/T		0/1
NM_001142800:c.-333+48921dupA	6:66300750	-/T		0/1
NM_001142800:c.-333+48921delA	6:66300750	T/-		REF
NM_001142800:c.-333+48659G>A	6:66301012	C/T	rs62407719	REF
NM_001142800:c.-333+48500A>G	6:66301171	T/C	rs72882209	ALT
NM_001142800:c.-333+48461C>G	6:66301210	G/C	rs115835301	ALT
NM_001142800:c.-333+48447A>T	6:66301224	T/A	rs116265847	ALT
NM_001142800:c.-333+48441G>C	6:66301230	C/G	rs9363403	ALT
NM_001142800:c.-333+48228G>A	6:66301443	C/T	rs12199868	ALT
NM_001142800:c.-333+47764T>G	6:66301907	A/C	rs354362	ALT
NM_001142800:c.-333+47719T>C	6:66301952	A/G	rs9351513	REF
NM_001142800:c.-333+46534A>G	6:66303137	T/C	rs16896892	REF
NM_001142800:c.-333+45977C>T	6:66303694	G/A	rs354363	ALT
NM_001142800:c.-333+45508A>G	6:66304163	T/C	rs354364	REF
NM_001142800:c.-333+45211A>G	6:66304460	T/C	rs354365	ALT
NM_001142800:c.-333+45155A>G	6:66304516	T/C		REF
NM_001142800:c.-333+44604T>A	6:66305067	A/T	rs13215741	REF
NM_001142800:c.-333+44308delT	6:66305363	A/-	rs200857384,rs:	0/.
NM_001142800:c.-333+44308T>A	6:66305363	A/T	rs200857384,rs:	0/1
NM_001142800:c.-333+44128dupT	6:66305543	-/A	rs61686102	0/1
NM_001142800:c.-333+43773T>C	6:66305898	A/G	rs9354267	ALT
NM_001142800:c.-333+43435dupA	6:66306236	-/T	rs11443686	ALT
NM_001142800:c.-333+42528T>C	6:66307143	A/G	rs2183675	ALT
NM_001142800:c.-333+41716A>G	6:66307955	T/C	rs189550	REF
NM_001142800:c.-333+41544T>G	6:66308127	A/C		0/1

Table S6

NM_001142800:c.-333+41540T>G	6:66308131	A/C	rs112829973	0/1
NM_001142800:c.-333+41529delT	6:66308142	A/-	rs354341	0/.
NM_001142800:c.-333+41529T>G	6:66308142	A/C	rs354341	0/1
NM_001142800:c.-333+41512G>T	6:66308159	C/A	rs35446336	REF
NM_001142800:c.-333+41346C>T	6:66308325	G/A	rs354342	REF
NM_001142800:c.-333+40579G>A	6:66309092	C/T	rs58886551	REF
NM_001142800:c.-333+40543A>T	6:66309128	T/A	rs57391513	REF
NM_001142800:c.-333+40356A>G	6:66309315	T/C	rs11963452	REF
NM_001142800:c.-333+40042G>A	6:66309629	C/T	rs77154807	REF
NM_001142800:c.-333+39983T>C	6:66309688	A/G	rs354343	ALT
NM_001142800:c.-333+39754G>A	6:66309917	C/T	rs17699558	ALT
NM_001142800:c.-333+39364G>T	6:66310307	C/A	rs12206843	ALT
NM_001142800:c.-333+39185G>A	6:66310486	C/T	rs16896896	REF
NM_001142800:c.-333+36525C>T	6:66313146	G/A	rs9453339	REF
NM_001142800:c.-333+35789G>A	6:66313882	C/T	rs168514	ALT
NM_001142800:c.-333+35748G>A	6:66313923	C/T	rs1929323	ALT
NM_001142800:c.-333+35645T>G	6:66314026	A/C	rs1387793	ALT
NM_001142800:c.-333+35521A>G	6:66314150	T/C	rs16896908	REF
NM_001142800:c.-333+35242A>C	6:66314429	T/G	rs6919704	ALT
NM_001142800:c.-333+34935A>G	6:66314736	T/C	rs11313675	REF
NM_001142800:c.-333+34791T>C	6:66314880	A/G	rs354401	ALT
NM_001142800:c.-333+34768dupA	6:66314903	-/T	rs10626958	0/.
NM_001142800:c.-333+34768delA	6:66314903	T/-		REF
NM_001142800:c.-333+34456delA	6:66315215	T/-	rs34132189	REF
NM_001142800:c.-333+34188A>G	6:66315483	T/C	rs181129415	REF
NM_001142800:c.-333+34080A>C	6:66315591	T/G	rs354400	REF
NM_001142800:c.-333+33938C>T	6:66315733	G/A		REF
NM_001142800:c.-333+33750A>G	6:66315921	T/C	rs6930884	ALT
NM_001142800:c.-333+33523G>T	6:66316148	C/A	rs1907018	ALT
NM_001142800:c.-333+33444G>A	6:66316227	C/T	rs354398	ALT
NM_001142800:c.-333+33308T>G	6:66316363	A/C	rs1907017	ALT
NM_001142800:c.-333+33211T>A	6:66316460	A/T	rs1907016	ALT
NM_001142800:c.-333+33129G>T	6:66316542	C/A	rs1907015	ALT
NM_001142800:c.-333+32893dupA	6:66316778	-/T	rs140167236	1/.
NM_001142800:c.-333+32893delA	6:66316778	T/-	rs5876976	REF
NM_001142800:c.-333+32813C>T	6:66316858	G/A	rs354397	ALT
NM_001142800:c.-333+32780A>G	6:66316891	T/C	rs354396	ALT
NM_001142800:c.-333+32692A>G	6:66316979	T/C	rs354395	ALT
NM_001142800:c.-333+32334A>G	6:66317337	T/C	rs354394	ALT
NM_001142800:c.-333+32304G>A	6:66317367	C/T	rs7748738	ALT
NM_001142800:c.-333+31531T>A	6:66318140	A/T	rs354392	ALT
NM_001142800:c.-333+31086G>A	6:66318585	C/T	rs10447346	ALT
NM_001142800:c.-333+30946T>G	6:66318725	A/C	rs10447316	ALT

Table S6

NM_001142800:c.-333+30441dupT	6:66319230	-/A	rs34304600	0/1
NM_001142800:c.-333+30400A>T	6:66319271	T/A	rs7744893	ALT
NM_001142800:c.-333+30191A>C	6:66319480	T/G	rs354391	ALT
NM_001142800:c.-333+29854A>T	6:66319817	T/A	rs7745746	ALT
NM_001142800:c.-333+29768G>A	6:66319903	C/T	rs1027188	ALT
NM_001142800:c.-333+29674A>C	6:66319997	T/G	rs35472556	REF
NM_001142800:c.-333+29501G>A	6:66320170	C/T	rs2351267	ALT
NM_001142800:c.-333+29332A>G	6:66320339	T/C	rs1490143	ALT
NM_001142800:c.-333+29152G>T	6:66320519	C/A	rs1490142	ALT
NM_001142800:c.-333+28901A>G	6:66320770	T/C	rs354387	ALT
NM_001142800:c.-333+28799C>T	6:66320872	G/A	rs191001672	REF
NM_001142800:c.-333+28599dupT	6:66321072	-/A	rs200048825	ALT
NM_001142800:c.-333+28545A>T	6:66321126	T/A	rs12198998	ALT
NM_001142800:c.-333+28521G>A	6:66321150	C/T	rs354386	ALT
NM_001142800:c.-333+27625T>C	6:66322046	A/G	rs12199458	ALT
NM_001142800:c.-333+27555T>C	6:66322116	A/G	rs183631341	REF
NM_001142800:c.-333+27548A>G	6:66322123	T/C	rs12200913	ALT
NM_001142800:c.-333+27090G>A	6:66322581	C/T	rs7747445	ALT
NM_001142800:c.-333+27006dupT	6:66322665	-/A	rs11385339	ALT
NM_001142800:c.-333+26470G>A	6:66323201	C/T	rs13201651	REF
NM_001142800:c.-333+26464G>A	6:66323207	C/T	rs10806512	ALT
NM_001142800:c.-333+26261C>T	6:66323410	G/A	rs354382	ALT
NM_001142800:c.-333+25792C>T	6:66323879	G/A	rs2130498	ALT
NM_001142800:c.-333+25678A>C	6:66323993	T/G	rs1980987	ALT
NM_001142800:c.-333+25409T>G	6:66324262	A/C	rs7739112	ALT
NM_001142800:c.-333+25311C>G	6:66324360	G/C	rs168512	ALT
NM_001142800:c.-333+25297dupA	6:66324374	-/T	rs35160142	REF
NM_001142800:c.-333+25176C>T	6:66324495	G/A	rs354381	ALT
NM_001142800:c.-333+24894_-333+24895insT	6:66324777	-/A	rs5876977	ALT
NM_001142800:c.-333+24879G>A	6:66324792	C/T	rs7758595	ALT
NM_001142800:c.-333+24652A>T	6:66325019	T/A	rs971620	REF
NM_001142800:c.-333+24033A>G	6:66325638	T/C	rs2046837	REF
NM_001142800:c.-333+24028G>A	6:66325643	C/T	rs2046836	REF
NM_001142800:c.-333+23938C>T	6:66325733	G/A	rs2046835	REF
NM_001142800:c.-333+23789A>T	6:66325882	T/A	rs12208402	ALT
NM_001142800:c.-333+23372delT	6:66326299	A/-	rs66800400	ALT
NM_001142800:c.-333+23067C>G	6:66326604	G/C	rs421733	REF
NM_001142800:c.-333+23063C>G	6:66326608	G/C	rs12213513	ALT
NM_001142800:c.-333+22994dupT	6:66326677	-/A	rs34322899	REF
NM_001142800:c.-333+22986delT	6:66326685	A/-		REF
NM_001142800:c.-333+22726A>G	6:66326945	T/C	rs13207080	REF
NM_001142800:c.-333+22654C>G	6:66327017	G/C	rs973300	REF
NM_001142800:c.-333+22641G>C	6:66327030	C/G	rs973299	ALT

Table S6

NM_001142800:c.-333+22620C>T	6:66327051	G/A	rs2351268	ALT
NM_001142800:c.-333+22501G>A	6:66327170	C/T	rs2351269	ALT
NM_001142800:c.-333+22039A>G	6:66327632	T/C	rs12210732	ALT
NM_001142800:c.-333+22025G>T	6:66327646	C/A	rs10944817	ALT
NM_001142800:c.-333+18242G>C	6:66331429	C/G	rs168511	REF
NM_001142800:c.-333+18158A>G	6:66331513	T/C	rs373700804	REF
NM_001142800:c.-333+18062T>C	6:66331609	A/G	rs187126	ALT
NM_001142800:c.-333+17802A>C	6:66331869	T/G	rs4298329	ALT
NM_001142800:c.-333+17323G>A	6:66332348	C/T	rs354371	ALT
NM_001142800:c.-333+17022delA	6:66332649	T/-	rs35201653	./1
NM_001142800:c.-333+16967T>C	6:66332704	A/G	rs354370	ALT
NM_001142800:c.-333+16771A>G	6:66332900	T/C	rs6926767	ALT
NM_001142800:c.-333+16566G>A	6:66333105	C/T	rs6900513	ALT
NM_001142800:c.-333+16152T>A	6:66333519	A/T	rs354369	ALT
NM_001142800:c.-333+16049G>C	6:66333622	C/G	rs728880	ALT
NM_001142800:c.-333+15999C>T	6:66333672	G/A	rs902287	ALT
NM_001142800:c.-333+15945G>C	6:66333726	C/G	rs728881	ALT
NM_001142800:c.-333+15397C>G	6:66334274	G/C	rs902286	ALT
NM_001142800:c.-333+15344C>T	6:66334327	G/A	rs4446546	ALT
NM_001142800:c.-333+14811A>T	6:66334860	T/A	rs12215492	ALT
NM_001142800:c.-333+14207C>T	6:66335464	G/A	rs1490139	ALT
NM_001142800:c.-333+14047T>A	6:66335624	A/T	rs354367	ALT
NM_001142800:c.-333+13705C>T	6:66335966	G/A	rs766199	ALT
NM_001142800:c.-333+13273C>T	6:66336398	G/A	rs6917909	ALT
NM_001142800:c.-333+13204G>T	6:66336467	C/A	rs6919315	ALT
NM_001142800:c.-333+13154A>T	6:66336517	T/A	rs968893	ALT
NM_001142800:c.-333+12908A>G	6:66336763	T/C	rs6903740	ALT
NM_001142800:c.-333+12872G>T	6:66336799	C/A	rs6919950	REF
NM_001142800:c.-333+12351T>C	6:66337320	A/G	rs9453347	ALT
NM_001142800:c.-333+12327G>A	6:66337344	C/T	rs139174916	REF
NM_001142800:c.-333+12079C>T	6:66337592	G/A	rs12197389	ALT
NM_001142800:c.-333+12029C>T	6:66337642	G/A	rs111411564	REF
NM_001142800:c.-333+11998G>A	6:66337673	C/T	rs12211326	ALT
NM_001142800:c.-333+11893G>A	6:66337778	C/T	rs12211385	ALT
NM_001142800:c.-333+11204C>T	6:66338467	G/A	rs6930269	ALT
NM_001142800:c.-333+11092T>C	6:66338579	A/G	rs62407730	REF
NM_001142800:c.-333+10904A>G	6:66338767	T/C	rs10455583	ALT
NM_001142800:c.-333+10823C>T	6:66338848	G/A	rs10455188	ALT
NM_001142800:c.-333+10537A>G	6:66339134	T/C	rs76385824	REF
NM_001142800:c.-333+10511C>G	6:66339160	G/C	rs10455189	ALT
NM_001142800:c.-333+10166T>C	6:66339505	A/G	rs12195089	ALT
NM_001142800:c.-333+9468G>A	6:66340203	C/T	rs1873292	ALT
NM_001142800:c.-333+9239G>A	6:66340432	C/T	rs2130493	ALT
NM_001142800:c.-333+9192A>G	6:66340479	T/C	rs10944827	ALT
NM_001142800:c.-333+9092G>A	6:66340579	C/T	rs10944828	ALT
NM_001142800:c.-333+8453A>G	6:66341218	T/C	rs1387800	ALT
NM_001142800:c.-333+8302dupT	6:66341369	-/A	rs5876981	0/1
NM_001142800:c.-333+7859C>A	6:66341812	G/T	rs6916915	ALT
NM_001142800:c.-333+7725G>A	6:66341946	C/T	rs16896967	REF

Table S6

NM_001142800:c.-333+7615A>C	6:66342056	T/G	rs13195796	REF
NM_001142800:c.-333+6998A>G	6:66342673	T/C	rs7745805	REF
NM_001142800:c.-333+5471G>C	6:66344200	C/G	rs10944829	ALT
NM_001142800:c.-333+4816G>A	6:66344855	C/T	rs1873291	REF
NM_001142800:c.-333+4680A>T	6:66344991	T/A	rs7760787	ALT
NM_001142800:c.-333+4573G>A	6:66345098	C/T	rs79739949	REF
NM_001142800:c.-333+4522G>A	6:66345149	C/T	rs1907010	ALT
NM_001142800:c.-333+4382C>T	6:66345289	G/A	rs4621617	ALT
NM_001142800:c.-333+3984C>T	6:66345687	G/A	rs7775327	ALT
NM_001142800:c.-333+3938G>T	6:66345733	C/A	rs6899544	ALT
NM_001142800:c.-333+3732G>A	6:66345939	C/T	rs6904116	ALT
NM_001142800:c.-333+3582C>A	6:66346089	G/T	rs1586656	ALT
NM_001142800:c.-333+3555A>G	6:66346116	T/C	rs138554316	REF
NM_001142800:c.-333+3540C>G	6:66346131	G/C	rs1586657	ALT
NM_001142800:c.-333+3483T>A	6:66346188	A/T	rs10944830	ALT
NM_001142800:c.-333+3324T>G	6:66346347	A/C	rs1586658	ALT
NM_001142800:c.-333+3081A>G	6:66346590	T/C	rs1490126	ALT
NM_001142800:c.-333+2789A>T	6:66346882	T/A	rs4458657	ALT
NM_001142800:c.-333+2735A>C	6:66346936	T/G	rs2046838	ALT
NM_001142800:c.-333+2726A>G	6:66346945	T/C	rs2046839	ALT
NM_001142800:c.-333+2170T>G	6:66347501	A/C	rs6933242	ALT
NM_001142800:c.-333+1849G>A	6:66347822	C/T	rs6911669	ALT
NM_001142800:c.-333+1760C>T	6:66347911	G/A	rs12207870	ALT
NM_001142800:c.-333+1506C>T	6:66348165	G/A	rs6915285	ALT
NM_001142800:c.-333+1292G>A	6:66348379	C/T	rs12195141	ALT
NM_001142800:c.-333+1049G>A	6:66348622	C/T	rs6917401	ALT
NM_001142800:c.-447-123A>C	6:66349908	T/G	rs17779372	ALT
NM_001142800:c.-447-529delA	6:66350314	T/-	rs11312384	ALT
NM_001142800:c.-447-1257C>A	6:66351042	G/T	rs7739824	ALT
NM_001142800:c.-447-1551C>T	6:66351336	G/A	rs72648382	ALT
NM_001142800:c.-447-2595C>G	6:66352380	G/C	rs6907955	ALT
NM_001142800:c.-447-2617A>T	6:66352402	T/A	rs3919983	ALT
NM_001142800:c.-447-2642delA	6:66352427	T/-	rs11299994	ALT
NM_001142800:c.-447-2753C>A	6:66352538	G/T	rs6908305	ALT
NM_001142800:c.-447-2816C>A	6:66352601	G/T	rs6908347	ALT
NM_001142800:c.-447-3877T>C	6:66353662	A/G	rs9363410	ALT
NM_001142800:c.-447-4228G>T	6:66354013	C/A	rs2351499	ALT
NM_001142800:c.-447-4603T>C	6:66354388	A/G	rs1826149	ALT
NM_001142800:c.-447-4641A>C	6:66354426	T/G	rs1826150	ALT
NM_001142800:c.-447-5024A>G	6:66354809	T/C	rs1994603	ALT
NM_001142800:c.-447-6123G>A	6:66355908	C/T	rs9363411	ALT
NM_001142800:c.-447-6804T>C	6:66356589	A/G	rs12207318	ALT
NM_001142800:c.-447-7019G>T	6:66356804	C/A	rs115460359	REF
NM_001142800:c.-447-7026T>A	6:66356811	A/T	rs10806514	ALT
NM_001142800:c.-447-7704T>C	6:66357489	A/G	rs9342492	ALT
NM_001142800:c.-447-7944T>G	6:66357729	A/C	rs6919287	ALT
NM_001142800:c.-447-8424C>T	6:66358209	G/A	rs112653175	REF
NM_001142800:c.-447-8426C>T	6:66358211	G/A		REF
NM_001142800:c.-447-8467T>C	6:66358252	A/G	rs56361312	ALT
NM_001142800:c.-447-8508A>C	6:66358293	T/G	rs1826151	ALT

Table S6

NM_001142800:c.-447-8816dupT	6:66358601	-/A	rs75589705	ALT
NM_001142800:c.-447-9018A>G	6:66358803	T/C	rs7765481	ALT
NM_001142800:c.-447-9038C>T	6:66358823	G/A	rs12190480	ALT
NM_001142800:c.-447-9071C>T	6:66358856	G/A	rs12191907	ALT
NM_001142800:c.-447-9246dupT	6:66359031	-/A	rs71677714	0/1
NM_001142800:c.-447-9275C>T	6:66359060	G/A	rs374536933	REF
NM_001142800:c.-447-9290A>T	6:66359075	T/A	rs372295707	REF
NM_001142800:c.-447-10184T>A	6:66359969	A/T	rs1387795	ALT
NM_001142800:c.-447-10443A>G	6:66360228	T/C	rs1387796	ALT
NM_001142800:c.-447-12600A>G	6:66362385	T/C	rs9345665	ALT
NM_001142800:c.-447-12982A>G	6:66362767	T/C	rs958705	ALT
NM_001142800:c.-447-13032G>A	6:66362817	C/T	rs958706	ALT
NM_001142800:c.-447-13214A>T	6:66362999	T/A	rs11758603	REF
NM_001142800:c.-447-15087dupT	6:66364872	-/A	rs36088825	REF
NM_001142800:c.-447-15820C>G	6:66365605	G/C	rs1844528	ALT
NM_001142800:c.-447-16373C>A	6:66366158	G/T	rs12195278	ALT
NM_001142800:c.-447-16546C>T	6:66366331	G/A	rs1602235	ALT
NM_001142800:c.-447-16662T>G	6:66366447	A/C	rs1602234	ALT
NM_001142800:c.-447-17970A>C	6:66367755	T/G	rs2200941	ALT
NM_001142800:c.-447-19519A>G	6:66369304	T/C	rs9345666	ALT
NM_001142800:c.-447-20781G>A	6:66370566	C/T	rs1387807	ALT
NM_001142800:c.-447-20994A>G	6:66370779	T/C	rs2171902	ALT
NM_001142800:c.-447-24071T>G	6:66373856	A/C	rs9345667	ALT
NM_001142800:c.-447-24072G>A	6:66373857	C/T	rs9363416	ALT
NM_001142800:c.-447-25019G>A	6:66374804	C/T	rs923537	ALT
NM_001142800:c.-447-25759A>C	6:66375544	T/G	rs7761627	ALT
NM_001142800:c.-447-25886T>A	6:66375671	A/T	rs7757773	ALT
NM_001142800:c.-447-26179dupA	6:66375964	-/T		REF
NM_001142800:c.-447-26179delA	6:66375964	T/-		REF
NM_001142800:c.-447-26209C>T	6:66375994	G/A	rs7741090	ALT
NM_001142800:c.-447-26281C>T	6:66376066	G/A	rs9354272	ALT
NM_001142800:c.-447-27066delT	6:66376851	A/-		REF
NM_001142800:c.-447-28089A>T	6:66377874	T/A	rs923534	ALT
NM_001142800:c.-447-28154A>G	6:66377939	T/C	rs923535	ALT
NM_001142800:c.-447-29854G>T	6:66379639	C/A	rs10455190	ALT
NM_001142800:c.-447-30126A>G	6:66379911	T/C	rs9283820	ALT
NM_001142800:c.-447-31642T>A	6:66381427	A/T	rs993015	ALT
NM_001142800:c.-447-32008A>G	6:66381793	T/C	rs1490120	ALT
NM_001142800:c.-447-32381T>C	6:66382166	A/G	rs7739376	ALT
NM_001142800:c.-447-33215C>T	6:66383000	G/A	rs12197906	ALT
NM_001142800:c.-447-33552T>C	6:66383337	A/G	rs6907252	ALT
NM_001142800:c.-448+33470G>A	6:66383558	C/T	rs6928284	ALT
NM_001142800:c.-448+32697T>C	6:66384331	A/G	rs10455585	ALT
NM_001142800:c.-448+30776T>G	6:66386252	A/C	rs7761171	ALT
NM_001142800:c.-448+30007C>T	6:66387021	G/A	rs200465206	REF
NM_001142800:c.-448+29090C>A	6:66387938	G/T	rs1907009	ALT
NM_001142800:c.-448+28329A>C	6:66388699	T/G	rs1490121	ALT

Table S6

NM_001142800:c.-448+28318dupT	6:66388710	-/A	rs34345454	0/1
NM_001142800:c.-448+28275C>T	6:66388753	G/A	rs1490122	ALT
NM_001142800:c.-448+26177G>A	6:66390851	C/T	rs2130491	ALT
NM_001142800:c.-448+25747C>T	6:66391281	G/A	rs2087538	ALT
NM_001142800:c.-448+24395C>G	6:66392633	G/C	rs1158424	ALT
NM_001142800:c.-448+23745A>T	6:66393283	T/A	rs117516822	REF
NM_001142800:c.-448+22870A>C	6:66394158	T/G	rs10944833	ALT
NM_001142800:c.-448+22430G>T	6:66394598	C/A	rs1490124	ALT
NM_001142800:c.-448+22266C>T	6:66394762	G/A	rs2130492	ALT
NM_001142800:c.-448+21737T>A	6:66395291	A/T	rs6904807	ALT
NM_001142800:c.-448+21499C>T	6:66395529	G/A	rs6924750	ALT
NM_001142800:c.-448+21324C>T	6:66395704	G/A	rs6924950	ALT
NM_001142800:c.-448+20795A>T	6:66396233	T/A	rs12208064	ALT
NM_001142800:c.-448+20646G>A	6:66396382	C/T	rs9354275	ALT
NM_001142800:c.-448+20345G>A	6:66396683	C/T	rs2351500	ALT
NM_001142800:c.-448+20048C>A	6:66396980	G/T	rs2351501	ALT
NM_001142800:c.-448+19541T>C	6:66397487	A/G	rs6455052	ALT
NM_001142800:c.-448+19517T>G	6:66397511	A/C	rs7754201	ALT
NM_001142800:c.-448+19337C>T	6:66397691	G/A		REF
NM_001142800:c.-448+19336G>C	6:66397692	C/G		REF
NM_001142800:c.-448+17964A>T	6:66399064	T/A		ALT
NM_001142800:c.-448+17183T>C	6:66399845	A/G	rs6902244	ALT
NM_001142800:c.-448+17131G>C	6:66399897	C/G	rs6923019	ALT
NM_001142800:c.-448+16406A>G	6:66400622	T/C	rs72648383	ALT
NM_001142800:c.-448+16232T>C	6:66400796	A/G	rs112562414	ALT
NM_001142800:c.-448+15354A>C	6:66401674	T/G	rs9445566	REF
NM_001142800:c.-448+14500T>C	6:66402528	A/G	rs35543758	ALT
NM_001142800:c.-448+13730delA	6:66403298	T/-	rs58238248	0/1
NM_001142800:c.-448+13191T>C	6:66403837	A/G	rs12211518	ALT
NM_001142800:c.-448+12992G>A	6:66404036	C/T	rs12204750	ALT
NM_001142800:c.-448+12846T>C	6:66404182	A/G	rs12529251	ALT
NM_001142800:c.-448+12733C>A	6:66404295	G/T	rs375062337	REF
NM_001142800:c.-448+12392dupT	6:66404636	-/A	rs34494006	0/1
NM_001142800:c.-448+12041C>T	6:66404987	G/A	rs12192822	ALT
NM_001142800:c.-448+11499delA	6:66405529	T/-	rs71815460	ALT
NM_001142800:c.-448+10943T>C	6:66406085	A/G	rs72648385	ALT
NM_001142800:c.-448+10270C>T	6:66406758	G/A	rs6916913	ALT
NM_001142800:c.-448+9020A>G	6:66408008	T/C	rs2812772	ALT
NM_001142800:c.-448+8980T>C	6:66408048	A/G	rs2812773	ALT
NM_001142800:c.-448+8824A>G	6:66408204	T/C	rs2636123	ALT
NM_001142800:c.-448+8553T>G	6:66408475	A/C	rs2812774	ALT
NM_001142800:c.-448+8270T>C	6:66408758	A/G	rs1873294	ALT
NM_001142800:c.-448+8058G>A	6:66408970	C/T	rs1873293	ALT
NM_001142800:c.-448+7273G>A	6:66409755	C/T	rs2812775	ALT
NM_001142800:c.-448+7244T>C	6:66409784	A/G	rs2812776	ALT
NM_001142800:c.-448+7239A>G	6:66409789	T/C	rs2814115	ALT

NM_001142800:c.-448+6942C>G		6:66410086	G/C	rs2812777	ALT
NM_001142800:c.-448+6328G>T		6:66410700	C/A	rs2812778	ALT
NM_001142800:c.-448+5697A>G		6:66411331	T/C	rs1490130	ALT
NM_001142800:c.-448+4330C>T		6:66412698	G/A	rs1387801	ALT
NM_001142800:c.-448+3949A>G		6:66413079	T/C	rs2814114	ALT
NM_001142800:c.-448+1760T>G		6:66415268	A/C	rs1021350	ALT
NM_001142800:c.-448+1338C>A		6:66415690	G/T	rs997075	ALT

RP1

HGVS c. (Clinically Relevant)	HGVS p. (Clinically Re	Chr:Pos	Ref/Alt	Identifier	Haplotype
NM_006269:c.-13+191A>G		8:55528953	A/G	rs702761	ALT
NM_006269:c.-13+311C>T		8:55529073	C/T	rs9643828	ALT
NM_006269:c.-13+725T>A		8:55529487	T/A	rs6473949	ALT
NM_006269:c.-13+850A>G		8:55529612	A/G	rs145290	ALT
NM_006269:c.-12-1998G>A		8:55531517	G/A	rs436527	ALT
NM_006269:c.788-202G>A		8:55537028	G/A		REF
NM_006269:c.788-193C>A		8:55537037	C/A		REF
NM_006269:c.788-92T>C		8:55537138	T/C	rs429668	ALT
NM_006269:c.2615G>A	p.Arg872His	8:55539057	G/A	rs444772	ALT
NM_006269:c.2690_2695delCTTTGA	p.Ser897Ter	8:55539132	CTTTGA/-		ALT
NM_006269:c.2953A>T	p.Asn985Tyr	8:55539395	A/T	rs2293869	REF
NM_006269:c.5008G>A	p.Ala1670Thr	8:55541450	G/A	rs446227	ALT
NM_006269:c.5071T>C	p.Ser1691Pro	8:55541513	T/C	rs414352	ALT
NM_006269:c.5175A>G	p.Gln1725=	8:55541617	A/G	rs441800	ALT
NM_006269:c.6098G>A	p.Cys2033Tyr	8:55542540	G/A	rs61739567	REF
NM_006269:c.*247_*248insTT		8:55543161	-/TT	rs10654889	REF
